# Gut Microbiome of an 11^th^ Century A.D. Pre-Columbian Andean Mummy

**DOI:** 10.1371/journal.pone.0138135

**Published:** 2015-09-30

**Authors:** Tasha M. Santiago-Rodriguez, Gino Fornaciari, Stefania Luciani, Scot E. Dowd, Gary A. Toranzos, Isolina Marota, Raul J. Cano

**Affiliations:** 1 Department of Pathology, University of California San Diego, San Diego, CA, United States of America; 2 Department of Translational Research on New Technologies in Medicine and Surgery, Division of Paleopathology, University of Pisa, Pisa, Italy; 3 Center for Anthropological, Paleopathological and Historical Studies of the Sardinian and Mediterranean Populations, Department of Biomedical Sciences, University of Sassari, Sassari, Italy; 4 Laboratory of Molecular Archaeo-Anthropology/ancient DNA, School of Biosciences and Veterinary Medicine, University of Camerino, Camerino, Italy; 5 Molecular Research LP (MR DNA), Shallowater, Texas, United States of America; 6 Department of Biology, University of Puerto Rico, San Juan, PR; 7 Center for Applications in Biotechnology, California Polytechnic State University, San Luis Obispo, CA, United States of America; University of Illinois at Urbana-Champaign, UNITED STATES

## Abstract

The process of natural mummification is a rare and unique process from which little is known about the resulting microbial community structure. In the present study, we characterized the microbiome of paleofeces, and ascending, transverse and descending colon of an 11^th^ century A.D. pre-Columbian Andean mummy by 16S rRNA gene high-throughput sequencing and metagenomics. Firmicutes were the most abundant bacterial group, with *Clostridium* spp. comprising up to 96.2% of the mummified gut, while *Turicibacter* spp. represented 89.2% of the bacteria identified in the paleofeces. Microbiome profile of the paleofeces was unique when compared to previously characterized coprolites that did not undergo natural mummification. We identified DNA sequences homologous to *Clostridium botulinum*, *Trypanosoma cruzi* and human papillomaviruses (HPVs). Unexpectedly, putative antibiotic-resistance genes including beta-lactamases, penicillin-binding proteins, resistance to fosfomycin, chloramphenicol, aminoglycosides, macrolides, sulfa, quinolones, tetracycline and vancomycin, and multi-drug transporters, were also identified. The presence of putative antibiotic-resistance genes suggests that resistance may not necessarily be associated with a selective pressure of antibiotics or contact with European cultures. Identification of pathogens and antibiotic-resistance genes in ancient human specimens will aid in the understanding of the evolution of pathogens as a way to treat and prevent diseases caused by bacteria, microbial eukaryotes and viruses.

## Introduction

Studies on the human microbiome represent an opportunity to better understand microbe-host interactions, the membership and ecology of microbes, and its impact in health and disease. The gut microbiome has been more extensively characterized compared to other human surfaces, and has been associated with several health conditions including obesity [1], colitis [2], autism [3], autoimmune diseases [4], cancer [5], diabetes [6] and inflammatory bowel disease [7]. Similar approaches have more recently been applied to characterize the microbial community structure of ancient samples [8–11]. This approach is augmenting our understanding of microbe-host interactions, and particularly, the evolution of commensal microorganisms and infectious diseases [9]. For instance, oral bacterial diversity has been shown to differ in modern subjects compared to those from the Neolithic Era, possibly due to changes in dietary habits [10]; and pathogens associated with oral diseases also have been identified in dental calculus [11]. Ancient microbiomes have also shown to act as reservoirs of putative antibiotic-resistance genes, indicating that antibiotic-resistance originates from ancient environments [11, 12].

Preservation of microbial DNA in ancient specimens requires specific conditions including freezing and rapid desiccation. For instance, amber formation, fossilization of fecal material and natural mummification preserve ancient microbes [8, 13, 14]. Amber is known to entrap diverse ancient bacteria and fungi, many of which may date to millions of years ago [14–16]. Fossilized fecal material or coprolites also harbor DNA from microbes known to inhabit the human gut, and microbiome profiles are distinguishable in ancient indigenous cultures [8]. However, the process of natural mummification and how microorganisms are preserved remains to be investigated in greater detail [9, 17, 18]. Natural mummification results from the combination of cold temperatures, low oxygen levels and dry conditions. Natural mummification requires the water content to decrease below a critical threshold, resulting in the inhibition of liquefying putrefaction by bacteria. Environmental conditions aid in tissue desiccation, where the body shrivels to a dry leathery mass of skin and tendons that surround the bone [19]. Naturally-preserved human mummies have been found in Africa, Europe, and North and South America, but few studies have investigated their microbial composition [9, 17, 20, 21].

The partial gut bacterial community profile of a pre-Columbian Andean mummy originating from Cuzco (Peru), the ancient capital of the Inca empire, was characterized by amplification of the 16S rRNA gene and revealed that *Clostridium* was among the most identifiable bacterial groups [17]. Moreover, a paleopathological study of this mummy demonstrated several different phenotypic abnormalities that suggest Chagas’ disease, caused by *Trypanosoma cruzi*, as a possible cause of death. A megavisceral syndrome including cardiomegaly, mega-oesophagus, gastric ectasia and megacolon with enormous amounts of feces was noted in the mummy. Other characteristics suggesting Chagas’ disease as a possible cause of death included massive fat substitution (*adipositas cordis*), with fibrosis of the myocardium, and fibrosis of oesophagus and colon [22, 23]. Notably, Chagas’ disease is endemic to central and South America, and transmission can occur though insects, contaminated blood, from the mother to the fetus, or by mucous membranes contaminated by feces containing the parasite [24]. Next-generation sequencing may be applied in combination with other techniques to augment information gathered from paleopathological analyses to trace pathogens associated with infectious diseases, including Chaga’s disease. This approach has also provided a reliable diagnosis of specific “modern” infectious diseases [25, 26].

The goals of the present study were to characterize the gut microbiome of a pre-Columbian Andean mummy using 16S rRNA gene high-throughput sequencing, and by utilizing metagenomics we sought to identify sequences homologous to *T*. *cruzi* and other potential pathogens, and identify and determine the evolution of genes associated with the identified pathogens.

## Materials and Methods

### Mummy description and paleopathology

**Fig 1:**
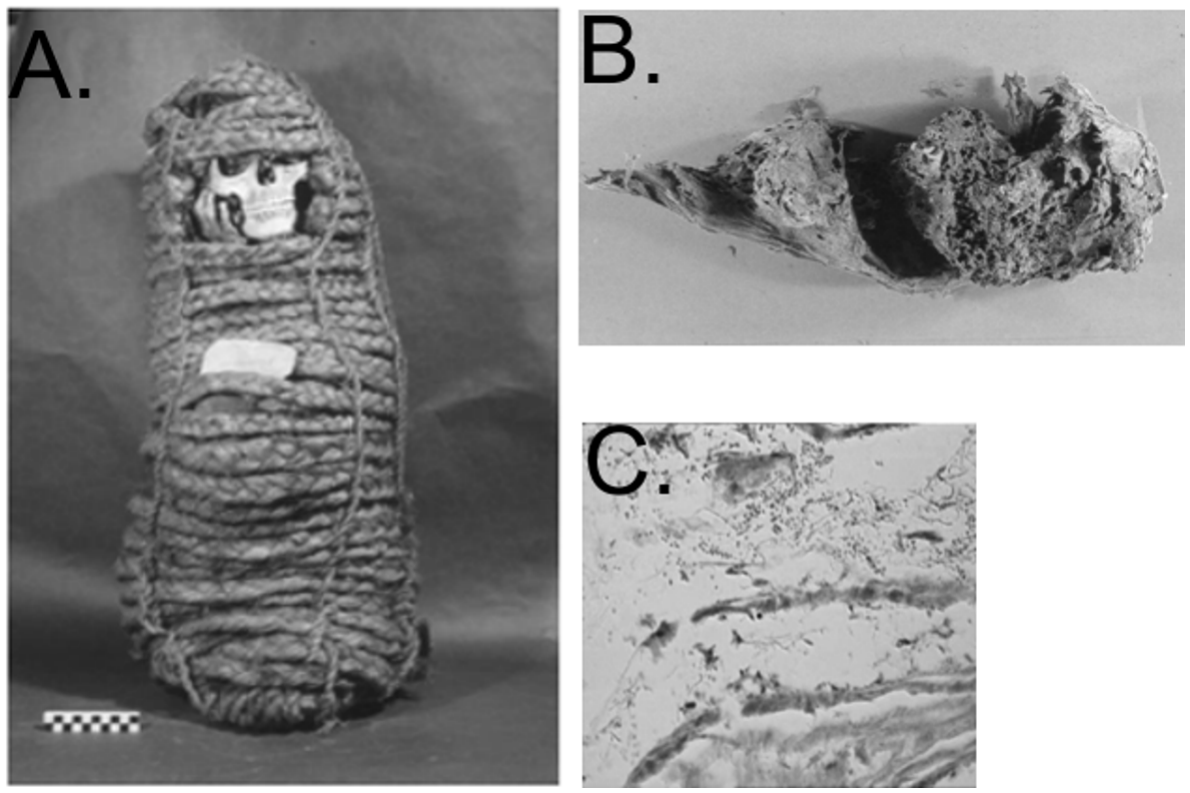
Pre-Columbian Andean mummy in this study (Panel A), distended colon with paleofeces (Panel B), and *Trypanosoma cruzi* amastigotes (Panel C).

The specimens utilized for DNA extraction were collected from a female pre-Columbian Andean mummy from Cuzco (Peru) with a 14C dating of 980–1170 A.D., presently stored at the Museum of Anthropology and Ethnology of the University of Florence, Italy, (catalogue number 3076). The body was brought from South America to Italy in the second half of the 19^th^ century by Professor Ernesto Mazzei. Autopsy was performed by paleopathologists G. Fornaciari and colleagues, and specimens were collected from internal organs [17, 27]. The mummy, of estimated age 18–23 years, lied inside a basket made of vegetal fibers (Fig 1A), which contained two drapes covering the body entirely. Only the head was found to be almost completely skeletonized. The mummy was found in fetal position, with ropes tied around the wrists, ankles and hips. The right posterior hemithorax was opened by cutting the skin tissues and the ribs.

The stomach was evidently ectasic and the esophagus seemed to be very enlarged. The left lung and the heart were then removed in block, revealing a severe cardiomegaly. A large amount of feces was present in the colon, which looked exceptionally distended (Fig 1B). The esophageal and cardiac tissues were previously stained with Giemsa, showing oval formations of about 1–2 μm (Fig 1C), with small nuclei. A previous immunohistochemical study with anti-flagellar *T*. *cruzi* antibody also showed a strong reactivity to immunoperoxidase in these small oval formations. Electron microscopy of the esophageal and colonic wall showed clusters of rare, irregularly oval formations, adherent to collagen fibers, of a maximum diameter of about 1 μm. Microscopic anatomy of sections of the heart was found to be markedly altered by *T*. *cruzi*. The colic wall, with fibrous structures and areas full of fecal material and colonies of amastigotes of *T*. *cruzi* were also observed. All these features are the characteristic appearance of amastigotes of the *Trypanosomatidae* family; therefore, it was concluded that the mummy was a case of Chagas’ disease in its chronic phase [27].

### Avoidance of DNA contamination and extraction

Paleofeces, or colon contents were collected directly from the descending colon during the autopsy, while colon samples were collected directly from tissue. Tissue samples to be used for DNA extraction were prepared from whole internal organs removed during the autopsy of the mummy and which had been stored aseptically. The autopsy was performed by paleopathologists wearing sterile surgical coats, sterile latex gloves, sterile masks, headdresses and overshoes. The mummified specimens were immediately kept and sealed in sterile plastic bags, reducing the opportunities for contamination. The outermost portions of the specimens were discarded to eliminate the risk of contamination and one replicate per sample type was obtained for further analyses. We employed the standard precautions for ancient DNA work including the use of sterile gloves, pretreatment of mortars, pestles, and homogenizers with HCl, use of UV-irradiated safety cabinets, dedicated gel trays, tanks and reagents. DNA extractions were conducted in the laboratory of Molecular Archaeo-Anthropology/ancient DNA in the University of Camerino (Italy), under strict rules to prevent DNA contamination. The ancient DNA laboratory was constructed exclusively for ancient DNA and no molecular analyses have ever been performed on modern DNA. The laboratory comprises an antechamber, in which the operator wears a full body sterile suit, gloves, a face screen, and an extraction room equipped with UV lights, and a positive-pressure air-filtering system providing 99.97% particle elimination (HEPA filtration system) and a complete change of air every 10 min.

We extracted DNA from approximately 0.2 g of paleofeces, and descending, transverse, and ascending colon using the phenol-chloroform method as described previously [17]. Briefly, samples were resuspended in an extraction medium composed of 50 mM Tris-HCl (pH 8.0), 50 mM Na_2_EDTA (pH 8.0), 1% (weight/volume) sodium dodecyl sulphate (SDS), and 6% (volume/volume) water-saturated phenol. Samples were left overnight at 4°C and transferred into sterile mortars and homogenized using sterile pestles. The homogenate was collected in Eppendorf tubes, taking care to rinse the mortar and pestle with extraction medium. The homogenates were sequentially extracted with equal volumes of phenol, phenol/chlorofom/isoamyl alcohol (25:24:1), chloroform/isoamyl alcohol (24:1) and ether, and the nucleic acid fraction was precipitated using ethanol at -20°C. Non template controls were added and processed following the same protocol in order to detect any potential contamination from reagents or the extraction process [17]. The quality of the DNA was checked in agarose gels, indicating that the DNA was fragmented and the size range of the fragments was between a dozen to 200–300 bp, consistent with the integrity of authentic ancient DNA [17].

### Analysis of 16S rRNA gene

DNA amplification of the 16S rRNA gene was performed at Molecular Research Laboratory (www.mrdnalab.com; Shallowater, TX, USA). All DNA samples were handled in exclusive areas for PCR amplification, which are sterilized before and after every use using DNAaway and UV-radiation to eliminate cross-contamination with modern samples. Template manipulations are handled in separate hoods that are sterilized before and after every manipulation using DNAaway and UV-radiation. Negative PCR controls were included in all amplification reactions. The 16S rRNA gene V4 variable region was amplified using the PCR primers 515f (GTGCCAGCMGCCGCGGTAA)/806r (GGACTACHVGGGTWTCTAAT). PCR amplifications were conducted using a single step 30 cycle PCR using the HotStarTaq Plus Master Mix Kit (Qiagen, USA) under the following conditions: 94°C for 3 minutes, followed by 28 cycles of 94°C for 30 seconds, 53°C for 40 seconds and 72°C for 1 minute, after which a final elongation step at 72°C for 5 minutes was performed. After amplification, PCR products were checked in 2% agarose gel to determine the success of amplification and the relative intensity of the bands. All amplicon products from each sample were mixed in equal concentrations and purified using Agencourt AMPure beads (Agencourt Bioscience Corporation, MA, USA). The pooled and purified PCR products were used to prepare the DNA library following Illumina MiSeq DNA library preparation protocol using the MiSeq reagent kit V3 (2X300 bp) for paired-end reads on a MiSeq following the manufacturer’s guidelines.

### Taxonomical analyses of bacterial communities and source-tracking

Fastq files corresponding to the mummy’s samples were joined using the QIIME pipeline using join_paired_ends.py [28]. Reads were assigned to samples based on their corresponding barcode using split_libraries.py with default filtering parameters. 16S rRNA sequence files were analyzed individually or merged with previously characterized coprolite microbiomes corresponding to the Saladoid and Huecoid pre-Columbian cultures for comparative purposes [8]. Saladoid and Huecoid coprolites were previously sequenced in the same facility and data were analyzed similarly to the mummy’s samples. 16S rRNA gene sequences were sorted based on sample ID using the QIIME script extract_seqs_by_sample_ id.py. De novo operational bacterial operational taxonomic units (OTUs) were selected using pick_de_novo_otus.py workflow. 16S rRNA taxonomy was defined by ≥97% similarity to reference sequences. Data was rarefied at 5,000 sequences per sample. The phylogenetic composition of the microcommunities present in the samples was characterized using summarize_taxa_through_plots.py up to the genus level. In order to assign presumptive bacterial species, BLASTn of the 16S rRNA gene sequences was performed against the NCBI 16S rRNA gene database downloaded using CLC Genomics Workbench 8.0 (CLC bio USA, Cambridge, MA, USA) with the following parameters: Match/Mismatch and Gap Costs = Match 2 Mismatch 3 Existence 5 Extension 2; Expectation value = 10.0; Filter low complexity = No, Maximum number of hits = 250; Number of threads = 1. Bayesian microbial source tracking was performed using SourceTracker to identify possible sources of contamination [9, 29]. Human sources included ten gut, saliva, and skin microbiomes, for a total of 30 sources, and were obtained from the Human Microbiome Project (http://hmpdacc.org/HMR16S/) (S1 File). Non-human sources included 45 soil microbiomes obtained from the SourceTracker tutorial (http://qiime.org/tutorials/source_tracking.html).

### Alpha rarefaction curves and diversity indices

Alpha rarefaction curves of the bacterial communities were computed using the alpha_rarefaction.py in QIIME. Alpha diversity metrics that included Phylogenetic Diversity (PD) whole tree, chao1 and observed species were plotted. Beta diversities were also computed using beta_diversity.py, with default parameters in QIIME. Procrustes plots were then constructed using transform_coordinate_matrices.py followed by make_emperor.py in QIIME. For comparative purposes, procrustes plots were constructed using coprolites from the Saladoid and Huecoid cultures as described previously [8], and human gut, saliva, and skin microbiomes downloaded from the Human Microbiome Project (http://hmpdacc.org/HMR16S/) (S1 File).

#### Metagenome analyses

DNA preparation for metagenome sequencing was also performed at Molecular Research Laboratory, (www.mrdnalab.com; Shallowater, TX, USA) under strict procedures to eliminate cross-contamination with modern DNA as described above. DNA library for metagenome analyses was prepared following the Illumina MiSeq DNA library preparation protocol using the MiSeq reagent kit V2 (2X150bp) for paired-end reads on a MiSeq following the manufacturer’s guidelines. Barcodes were trimmed using a proprietary analysis pipeline from MRDNA. Fastq files were then joined using CLC genomics workbench default parameters to join fastq files generated by the Illumina platform. Briefly, fastq files were imported into CLC Genomics Workbench as Paired-reads (where it is assumed that the first reads of the pairs are in one file and the second reads of the pairs to be in another), with a minimum and maximum distance of 50 and 250, respectively, with forward orientation and removing failed reads.

Paired reads were then used for all the metagenome analyses. We trimmed each read according to Phred- or Q-scores of 0.5, meaning that there is a 50% chance that a base may be incorrect, removed any low complexity reads with ≥8 consecutive homopolymers, and removed any reads with substantial length variation (<50 nucleotides) or ambiguous characters (N’s) prior to further analysis using CLC Genomics Workbench. Data were uploaded and annotated using the MG-RAST pipeline. Bray–Curtis indices from the taxonomic composition were retrieved from MG-RAST and were represented in a heatmap format to show the similarity and dissimilarity between microbial communities. The heatmap was constructed using R version 3.0.1 using the heatmap2 function. Categories were determined based on homologies to gene categories in the SEED database using the MG-RAST pipeline with a maximum e-value cutoff of 1.0 e^-5^, and a minimum identity cutoff of 80%.

We were interested in sequences homologous to C. *botulinum*, *T*. *cruzi*, and viruses (both bacteriophages and eukaryotic viruses). *Clostridium botulinum* was previously found to be significantly represented in the mummy’s gut, but results were limited to the technology available at the time [17]. We also were interested in the detection of sequences homologous to *T*. *cruzi* as a way to support paleopathological data strongly suggesting its presence in this mummy. As part of the mummy’s gut microbiome, we also were interested in sequences homologous to viruses as very few studies have focused on their presence in ancient samples [30]. To investigate this, reads were mapped to the genomes of *C*. *botulinum* strains NCTC 8266 (CP010520), NCTC 8550 (CP010521), and type B strain 111 (AP014696), and to *Clostridium botulinum* strain 111 plasmid (NC_025146.1) given that it carries important virulence factors including the botulinum neurotoxin (BoNT). Reads were also mapped to a virus database that included both prokaryotic and eukaryotic viruses (www.phantome.org; ftp://ftp.ncbi.nih.gov/genomes/Viruses/. Mapping was performed using CLC Genomics Workbench with the following parameters: no masking, mismatch cost = 2, insertion cost = 3, deletion cost = 3, with an 80% identity over a minimum of 50% of the read length. For the identification of sequences homologous to *T*. *cruzi*, we performed BLASTn against strains CL Brener, Dm28c, Esmeraldo, JR cl4, Marinkellei, Sylvio and Tula cl2 using contigs in order to obtain more significant matches. To generate the contigs, reads were assembled using CLC Genomics Workbench with the following parameters: Mapping mode = Map reads back to contigs (slow); Update contigs = Yes; Automatic bubble size = Yes; Minimum contig length = 100; Perform scaffolding = Yes; Auto-detect paired distances = Yes; Mismatch cost = 2; Insertion cost = 3; Deletion cost = 3 using an 80% identity over a 50% of the read length.

*Clostridium botulinum* plasmid integrase sequence from the mummified gut tissue was retrieved from the mapping analyses and BLASTn against the NCBInr database to confirm its identity. We reconstructed the phylogeny of the plasmid using the integrase sequence because these are essential for the integration of mobile genetic elements into the host’s genome [31]. The sequence was then aligned using CLC Genomics Workbench default parameters for multiple alignment with extant *C*. *botulinum* type B strain 111, *Clostridium autoethanogenum* DSM10061, *Clostridium ljungdahlii* DSM13528, and Alkaliphilus metalliredigens QYMF integrase sequences. Alignment was then used to reconstruct the phylogeny of the integrase genes using CLC Genomics Workbench default parameters for maximum likelihood phylogeny using Neighbor Joining, and Jukes Cantor distance with bootstrap resampling (100 replicates). Ribosomal RNA large subunit alpha corresponding to presumptive *T*. *cruzi* from the mummy was retrieved and aligned with sequences from modern *T*. *cruzi* strains CL Brener and Esmeraldo, and *Leishmania donovani* (GCA_000227135.2). Phylogenies were reconstructed using CLC Genomics Workbench default parameters for maximum likelihood phylogeny as described above. Mapped regions to human papillomaviruses (HPVs) types 21 (U31779.1) and 49 (NC_001541) were retrieved and BLASTn against the NCBInr database to confirm their identity. Sequences were then aligned and phylogenies were reconstructed using CLC Genomics Workbench default parameters for maximum likelihood phylogeny as described above.

Antibiotic-resistance genes have been found in ancient samples including the oral cavity and bacteriophages from a 14^th^ century European coprolite [10, 30]; yet, few studies have investigated the presence of antibiotic-resistance genes in ancient gut microbiomes, particularly in mummies [9, 12]. For the determination of the proportion of sequences associated with antibiotic-resistance, we performed BLASTx against the Comprehensive Antibiotic Resistance Database (CARDs) using the contigs in order to enable more productive searches for homologous sequences. We eliminated any homologues that could result in antibiotic resistance through mutation, including DNA topoisomerases, DNA gyrases, DNA polymerases, RNA polymerases, ribosomal RNA and ribosomal proteins. Homologues were classified according to the antibiotic classes beta lactamases, penicillin binding proteins, macrolides, tetracyclines, quinolones, sulfonamides, aminoglycosides, glycopeptides (vancomycin), chloramphenicol, fosfomycin, and multi-drug efflux pumps.

## Results

### 16S rRNA gene diversity of mummified gut tissue

**Fig 2:**
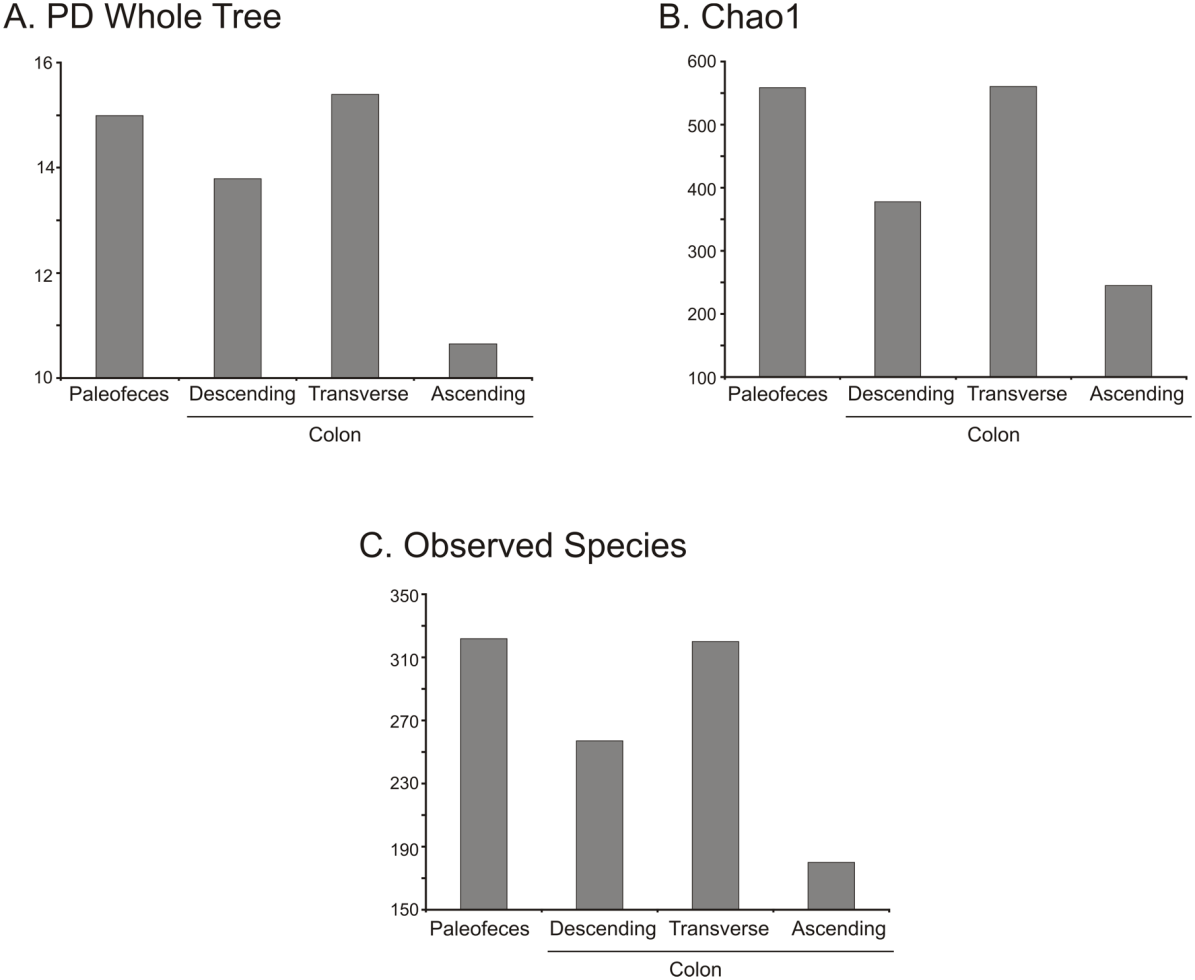
Bar plot representing the PD whole tree (Panel A), chao 1 (Panel B) and observed species (Panel C) indeces for the bacterial taxonomy based on 16S rRNA gene for paleofeces and mummified descending, transverse and ascending colon.

**Fig 3:**
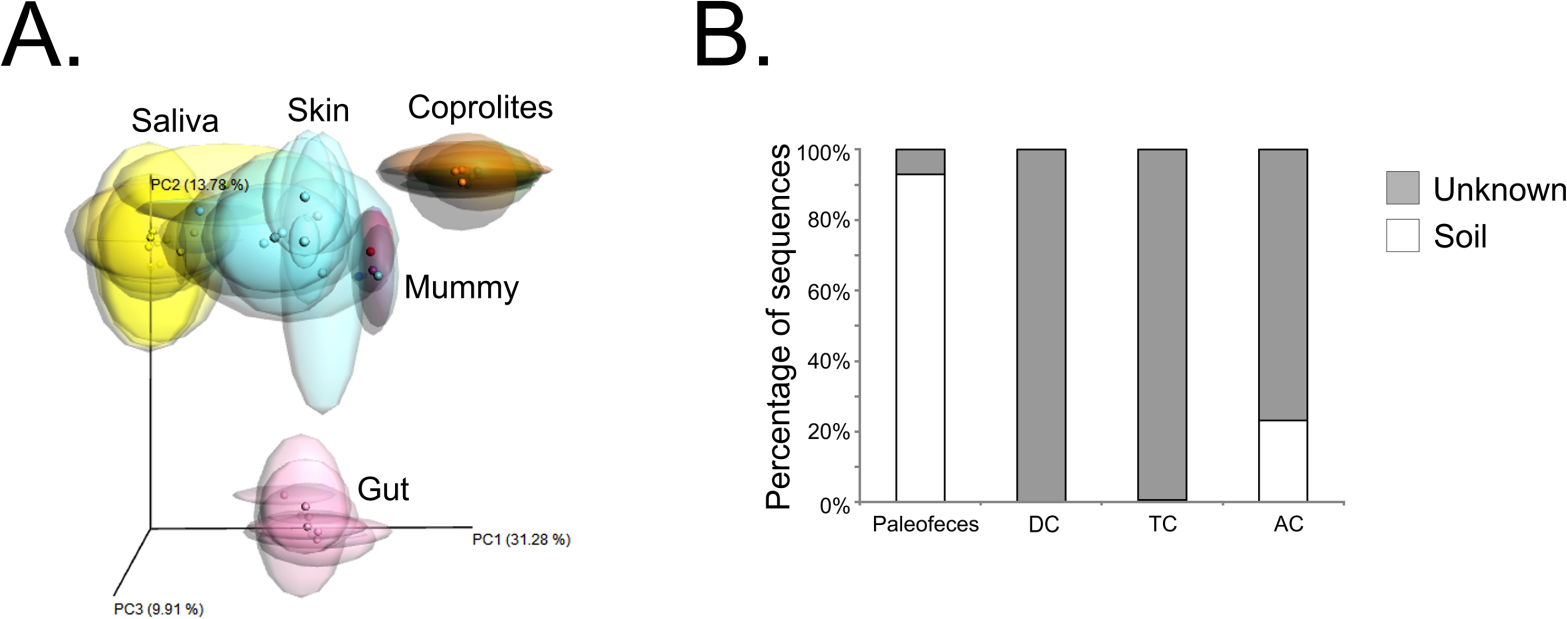
Panel A shows the procrustes analyses of extant human gut, saliva and skin, coprolites that did not undergo natural mummification, and mummy’s microbiomes. Panel B shows the Bayesian Source-Tracker results of paleofeces, descending colon (DC), transverse colon (TC) and ascending colon (AC) using soil microbiomes as non-human sources.

A total of 7,103 to 3,027,316 reads, with an average length of 270 bp and a GC-content percent ranging from 45.9 to 51.4% (Table 1) were analyzed. Alpha rarefaction curves for each sample type (n = 1) are shown in S1 Fig. We plotted alpha diversity measures present in bacterial communities using PD whole tree (Fig 2A), chao1 (Fig 2B) and observed species (Fig 2C). All alpha diversity indices showed that the paleofeces and transverse colon had the highest diversity, while the descending and ascending colon had the lowest. We also visualized beta-diversity measures of the paleofeces, and descending, transverse and ascending colon using procrustes plots. For comparison, we included the 16S rRNA gene profiles from ten pre-Columbian coprolites that did not undergo natural mummification and have previously been characterized using 16S rRNA gene high-throughput sequencing [8], as well as extant gut, saliva and skin microbiomes. We found that the majority of the human, coprolites and mummy microbiomes clustered according to sample type (Fig 3A). The mummy microbiomes shared some resemblance with the skin microbiomes, consistent with the source of the mummy’s sample collection being mainly the colon tissue. SourceTracker analyses with these same microbiomes showed that the mummy’s samples did not match any of the extant or coprolite microbiomes. In order to eliminate the possibility of non-human sources contributing to the results, we also performed the SourceTracker analyses using 45 soil microbiomes available in the SourceTracker tutorial [29]. Approximately 90% of the paleofeces microbiome matched soil microbiomes, while the vast majority of the descending, transverse and ascending colon microbiomes had no significant matches to any of the microbiomes used to track possible sources of contamination (Fig 3B).

**Table 1:** Sequence statistics.

**16S rRNA gene**			
**Sample (n = 1)**	**Number of reads**	**Average Length (bp)**	**Average GC Content (%)**
Paleofeces	399,101	270	51.4
Descending colon	8,731	270	50.2
Transverse colon	7,103	270	51.3
Ascending colon	3,027,316	270	45.9
**Metagenome**			
**Sample (n = 1)**	**Number of reads**	**Average Length (bp)**	**Average GC Content (%)**
Descending colon	146,081,692	100	48.0
Transverse colon	119, 843, 550	100	49.0
Ascending colon	140, 420, 752	100	50.0

Analysis of the 16S rRNA gene revealed that Firmicutes comprised 99.8%, 98.5%, 99.4% and 96.9% of the paleofeces, and descending, transverse and ascending colon microbiomes, respectively; while proteobacteria comprised 0.1%, 1.0%, 0.4% and 1.5% of paleofeces, and descending, transverse and ascending colon microbiomes, respectively. Other groups included the Actinobacteria and Cyanobacteria, which comprised <1.0% of the pre-Columbian Andean mummy gut microbiome. When analyzed at the family level, *Clostridiceae* comprised the majority of the paleofeces (58.8%), and descending (60.1%), transverse (96.9%) and ascending colon (95.4%) microbiomes. *Turicibacteraceae* was also among the highly represented families and comprised 29.8%, 0.5%, 1.9% and 12.4% of the paleofeces, and descending, transverse and ascending colon microbiomes, respectively.

**Fig 4:**
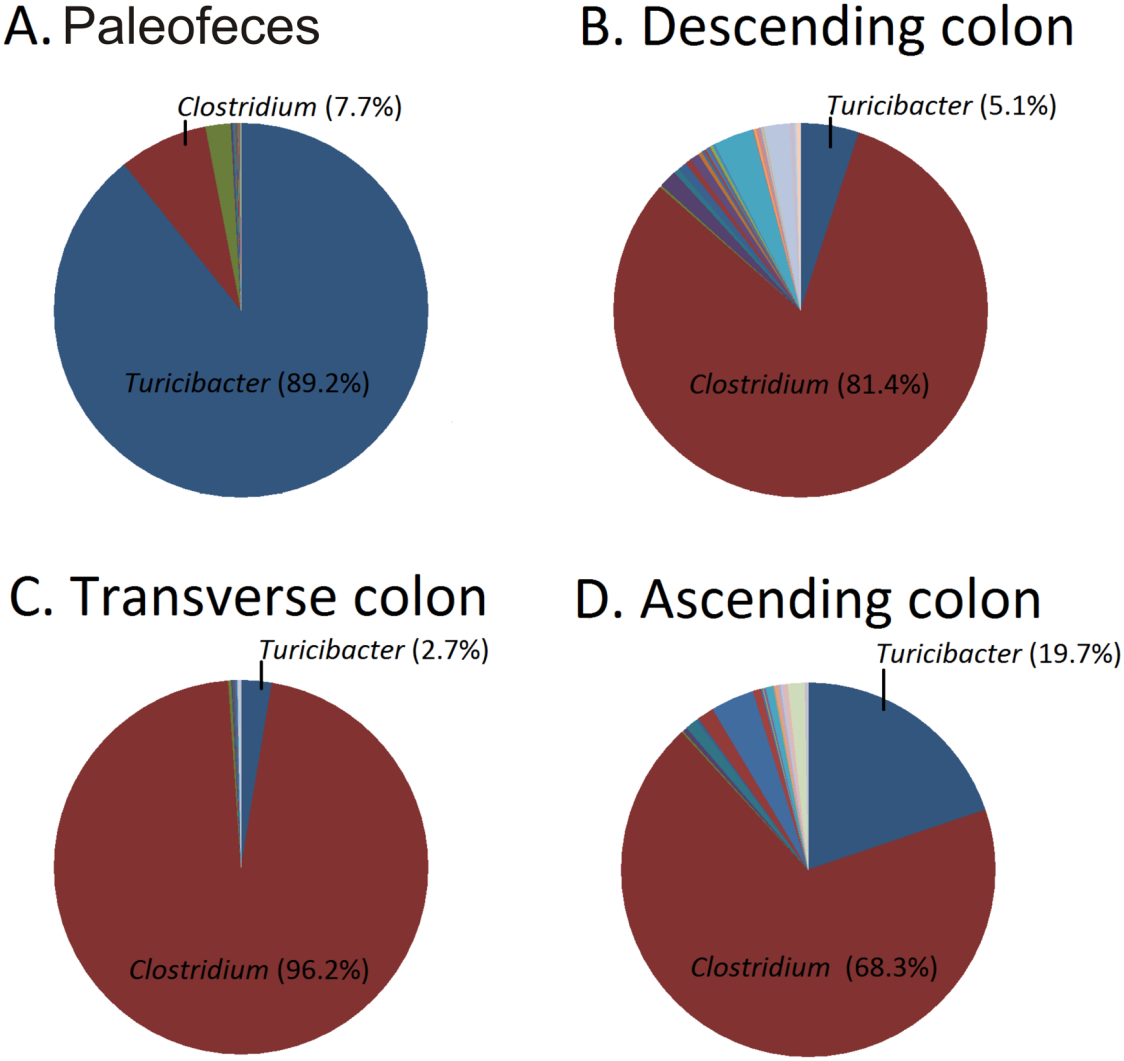
Pie charts representing bacterial taxonomy based on 16S rRNA gene at the genus level for paleofeces (Panel A), descending colon (Panel B), transverse colon (Panel C), and ascending colon (Panel D).

**Fig 5:**
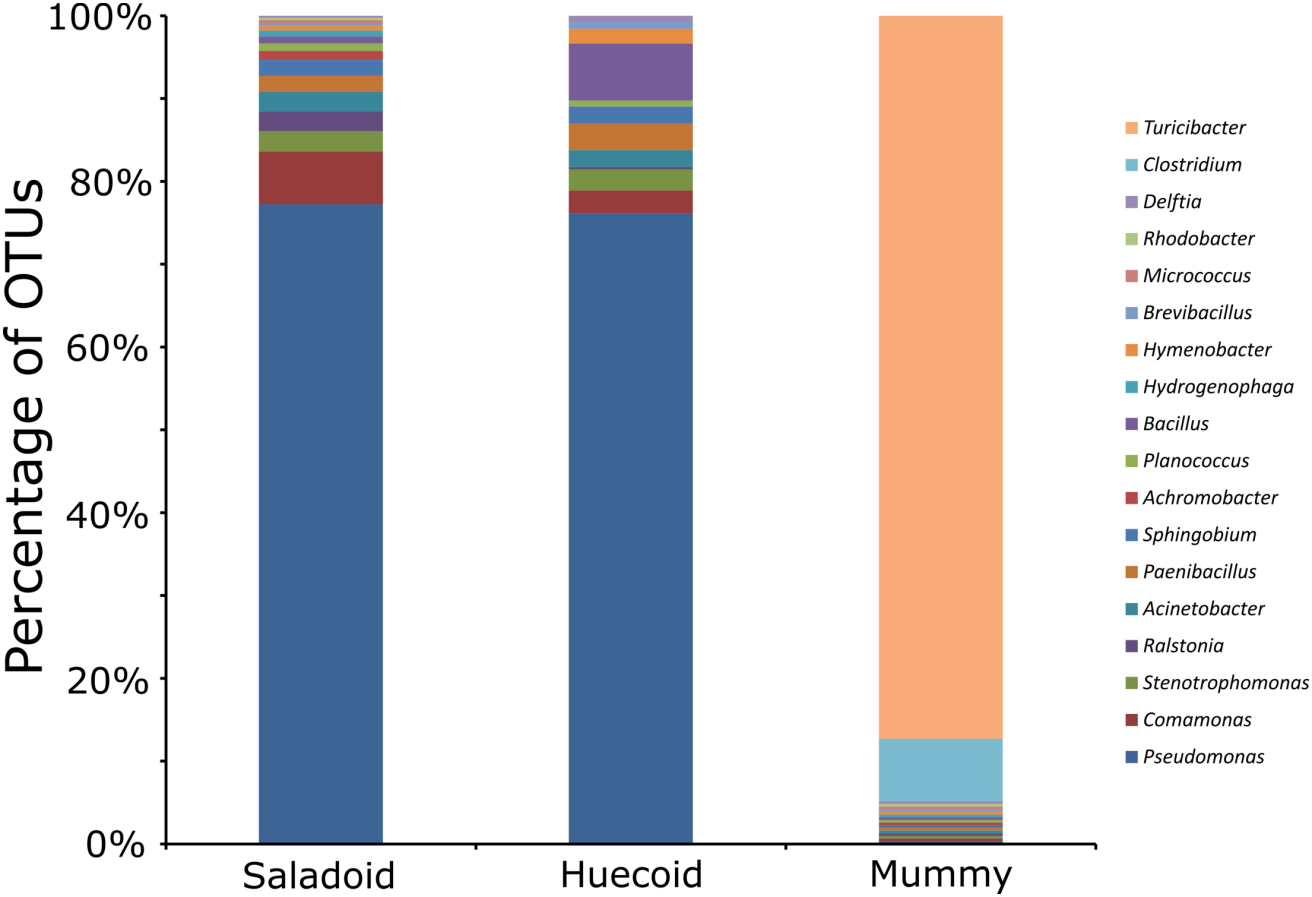
Bar charts representing bacterial taxonomy based on 16S rRNA gene at the genus level for coprolites that did not undergo natural mummification (Saladoid and Huecoid cultures) and the mummified paleofeces.

At the genus level, 66.7%, 90.2%, 27.7% and 37.6% of the paleofeces, and descending, transverse and ascending colon microbiomes could not be taxonomically assigned. Sequences that were taxonomically assigned at the genus level corresponded mostly to *Clostridium* and *Turicibacter* spp. (Fig 4). *Clostridium* spp. comprised 7.7%, 81.4%, 96.2% and 68.3% of the paleofeces (Fig 4A), and descending (Fig 4B), transverse (Fig 4C) and ascending colon (Fig 4D) microbiomes, respectively. All the taxonomical assignments at the genus level for the paleofeces, and descending, transverse and ascending colon are listed in S1 Table. The paleofeces were taxonomically compared with the mean relative abundances of coprolites that did not undergo natural mummification, corresponding to the Saladoid and Huecoid pre-Columbian cultures. The taxonomical composition of the pre-Columbian coprolites of the Saladoid and Huecoid cultures was distinct when compared to the paleofeces. *Pseudomonas* is among the most represented genus in the Saladoid and Huecoid coprolites, while *Turicibacter* is well represented in the mummy’s paleofeces and was not identified in the Saladoid or Huecoid coprolites (Fig 5).

We used BLASTn against the NCBI database to annotate 16S rRNA gene sequences at the species level. Several of the best hits found in the paleofeces, and descending, transverse and ascending colon included *Clostridium tetani*, *Clostridium sporogenes*, *Clostridium disporicum*, *Clostridium tertium*, *Clostridium bifermentans* and *Clostridium difficile*. Several other species where unique to each mummified specimen and were not restricted to spore-forming bacteria (Table 2). Presumptive bacterial species identified in the paleofeces, descending, transverse and ascending colon using BLASTn against the NCBI 16S rRNA gene database are shown in S2, S3, S4 and S5 Tables along with their identity percentages, e-values and accession numbers for the best hits.

**Table 2:** Several presumptive bacterial species identified in the Pre-Columbian Andean mummy paleofeces, and descending, transverse and ascending colon based on BLASTn analyses.

Bacteria	Paleofeces	Descending colon	Transverse colon	Ascending colon
*Acinetobacter johnsonii*	-	+	+	-
*Aggregatibacter aphrophilus*	-	+	-	-
*Bacillus circulans*	+	-	-	-
*Bacillus idriensis*	-	+	-	-
*Bacillus toyonensis*	+	-	-	-
*Bacteroides stercorirosoris*	+	-	-	-
*Clostridium aldenense*	+	-	+	-
*Clostridium algidicarnis*	+	-	+	-
*Clostridium bifermentans*	+	+	-	-
*Clostridium butyricum*	+	-	+	-
*Clostridium chromiireducens*	-	+	+	-
*Clostridium cochlearium*	-	-	-	+
*Clostridium colinum*	+	-	-	-
*Clostridium cylindrosporum*	-	-	+	-
*Clostridium difficile*	+	-	-	-
*Clostridium disporicum*	+	+	+	+
*Clostridium estertheticum*	-	-	-	+
*Clostridium indolis*	-	+	+	-
*Clostridium jejuense*	-	-	-	+
*Clostridium lavalense*	-	-	-	+
*Clostridium methylpentosum*	-	+	+	-
*Clostridium paraputrificum*	+	-	-	-
*Clostridium peptidivorans*	-	+	-	-
*Clostridium puniceum*	-	-	+	-
*Clostridium putrefaciens*	+	+	+	+
*Clostridium quinii*	+	-	-	-
*Clostridium saccharobutylicum*	-	+	-	-
*Clostridium saccharogumia*	+	-	-	-
*Clostridium sardiniense*	-	-	+	-
*Clostridium septicum*	-	-	+	-
*Clostridium sordellii*	+	-	-	-
*Clostridium sporogenes*	-	+	-	-
*Clostridium tertium*	+	-	+	-
*Clostridium tetani*	-	+	+	+
*Clostridium uliginosum*	-	+	+	+
*Coprococcus eutactus*	+	-	-	-
*Corynebacterium coyleae*	-	-	+	-
*Corynebacterium terpenotabidum*	-	+	-	-
*Corynebacterium tuberculostearicum*	-	-	+	-
*Escherichia fergusonii*	-	-	-	+
*Eubacterium coprostanoligenes*	+	-	-	-
*Intestinibacter bartlettii*	-	+	+	+
*Lactobacillus leichmannii*	+	-	-	-
*Loriellopsis cavernicola*	-	-	-	+
*Neisseria cinerea*	-	+	-	-
*Pedobacter saltans*	-	-	-	+
*Prevotella loescheii*	-	-	+	-
*Prevotella melaninogenica*	-	+	-	-
*Pseudomonas aeruginosa*	-	-	-	+
*Robinsoniella peoriensis*	-	-	-	+
*Romboutsia ilealis*	+	+	+	-
*Ruminococcus bromii*	+	-	-	-
*Ruminococcus champanellensis*	+	-	-	-
*Sarcina maxima*	+	-	-	-
*Sarcina ventriculi*	+	-	-	-
*Shewanella baltica*	-	-	-	+
*Staphylococcus agnetis*	+	-	-	-
*Staphylococcus pasteuri*	+	-	-	-
*Streptococcus parasanguinis*	+	-	-	-
*Streptococcus thermophilus*	-	+	-	-
*Tissierella creatinophila*	-	+	+	+
*Tissierella praeacuta*	+	+	+	+
*Turicibacter sanguinis*	+	+	+	+

### Metagenome analysis reveals the presence of other taxa and ancient pathogens

**Fig 6:**
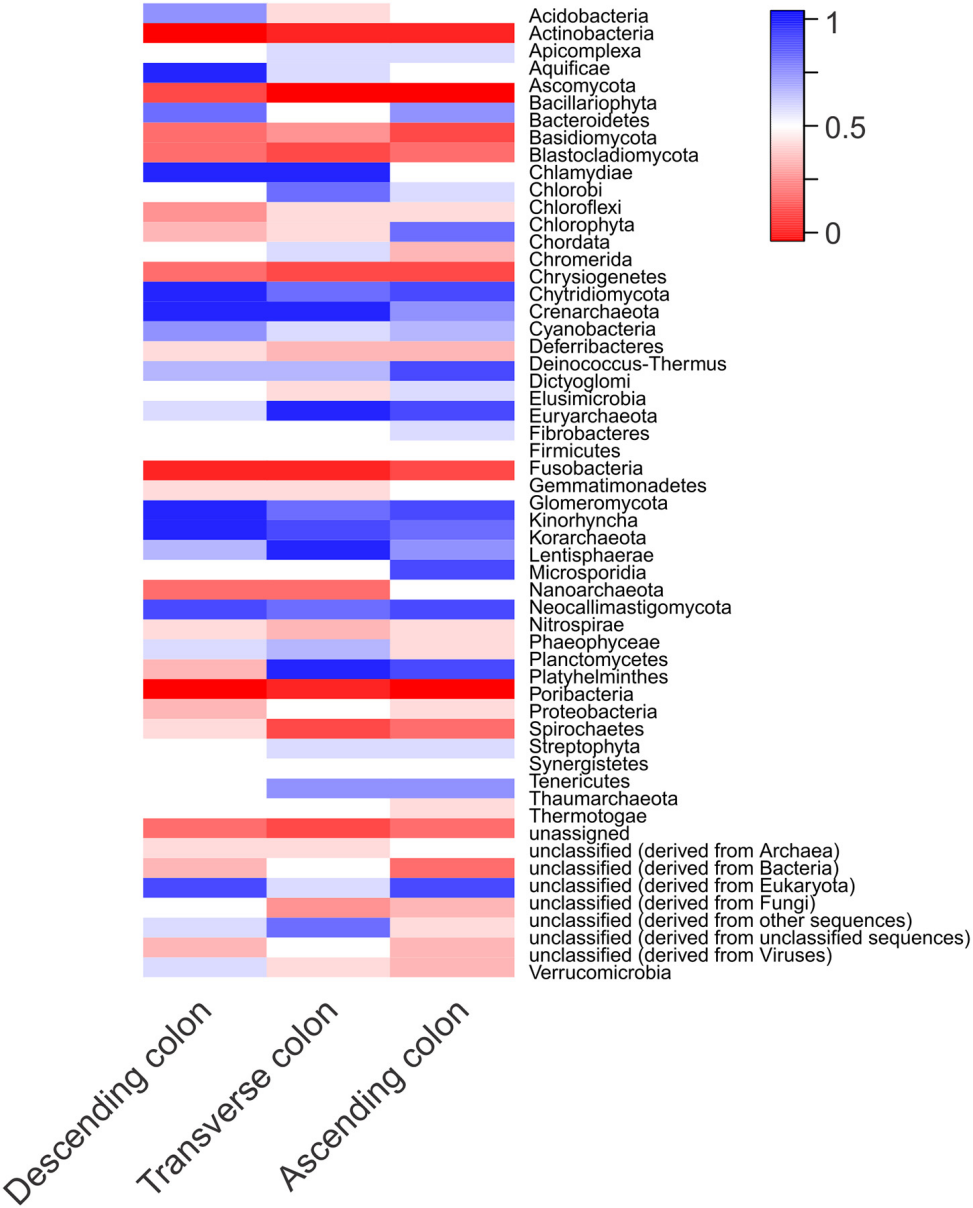
Heatmap of normalized bray-curtis distances of taxonomical assignments of descending, transverse and ascending colon. Values <0.5 are shown in red, values = 0.5 are shown in white and values >0.5 are shown in blue.

A total of 146,081, 692 (descending colon), 119, 843, 550 (transverse colon) and 140, 420, 752 (ascending colon) reads with an average length of 100 bp were generated for the metagenome analyses (Table 1). While we found bacterial communities using metagenomics that were not identified using the 16S rRNA gene, other sequences associated with archaea, fungi, viruses and eukaryotes were also identified. The heatmap in Fig 6 shows the normalized values of Bray-Curtis distances for each taxonomical category. Among the most abundant sequences were those associated with Firmicutes, Ascomycota, Proteobacteria, Actinobacteria, Bacteroidetes and Basidiomycota (Fig 6).

### Clostridium botulinum

**Fig 7:**
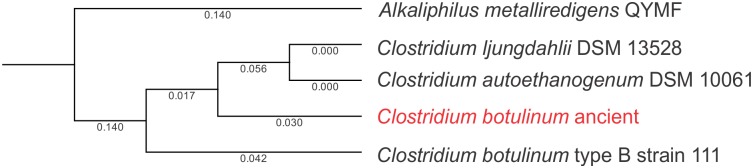
Phylogenetic reconstruction of ancient and extant *C*. *botulinum* type B strain 111 plasmid integrase, and plasmid integrases corresponding to *Alkaliphilus metalliredigens* QYMF, *C*. *ljungdahlii* DSM 13528, and *C*. *autoethanogenum* DSM 10061. Analyses were performed using maximum likelihood phylogeny using Neighbor Joining, and Jukes Cantor distance with bootstrap resampling (100 replicates). Branch lengths are shown.

Given that few reads (< 20) mapped to strains NCTC 8266 and NCTC 8550, and more reads mapped to the genome of *C*. *botulinum* type B strain 111 (52, 920 for descending colon; 39, 944 for transverse colon; 32, 784 for ascending colon), further analyses were performed with this strain. A total of 3,579 (descending colon), 3,089 (transverse colon) and 1,559 (ascending colon) reads mapped to *C*. *botulinum* type B strain 111 plasmid. To further validate the mapping, we performed BLASTn against the NCBInr database using the plasmid integrase sequence, confirming that this is indeed the best hit for the integrase (99% identity and e-value = 0.0). Sequence hits to other plasmid integrases were retrieved (S1 Dataset) and the phylogeny was reconstructed, revealing that the plasmid integrase from the ancient presumptive *C*. *botulinum* was similar to its modern counterpart (Fig 7).

### *Trypanosoma cruzi*-Chagas’ disease

**Fig 8:**
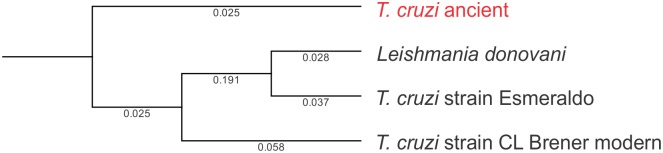
Phylogenetic reconstruction of large ribosomal subunit alpha from ancient *T*. *cruzi* and extant *T*. *cruzi* strains CL Brenner and Esmeraldo, and *Leishmania donovani*. Analyses were performed using maximum likelihood phylogeny using Neighbor Joining, and Jukes Cantor distance with bootstrap resampling (100 replicates). Branch lengths are shown.

Given the proportion of unclassified eukaryotic sequences in the mummified gut (Fig 6), we investigated the possibility that some may share homology with extant *T*. *cruzi* by performing BLASTn against seven known strains of *T*. *cruzi* (S2, S3, S4, S5, S6, S7 and S8 Datasets). BLASTn results were obtained from all seven strains and are summarized in S6 Table. The large ribosomal subunit alpha was then used for the phylogenetic analyses, with which we first performed BLASTn against the NCBInr database to validate this region, showing that the gene in presumptive ancient *T*. *cruzi* has a 90% identity with modern *T*. *cruzi* strain CL Brener (e-value = 6e^-30^). By comparing a partial sequence homologous to the large ribosomal subunit alpha of the presumptive ancient *T*. *cruzi* with modern strains CL Brenner and Esmeraldo, and *L*. *donovani*, we suggest that this pathogen may have a more remote origin than previously expected (Fig 8)(S9 Dataset).

### Human papillomaviruses

**Fig 9:**
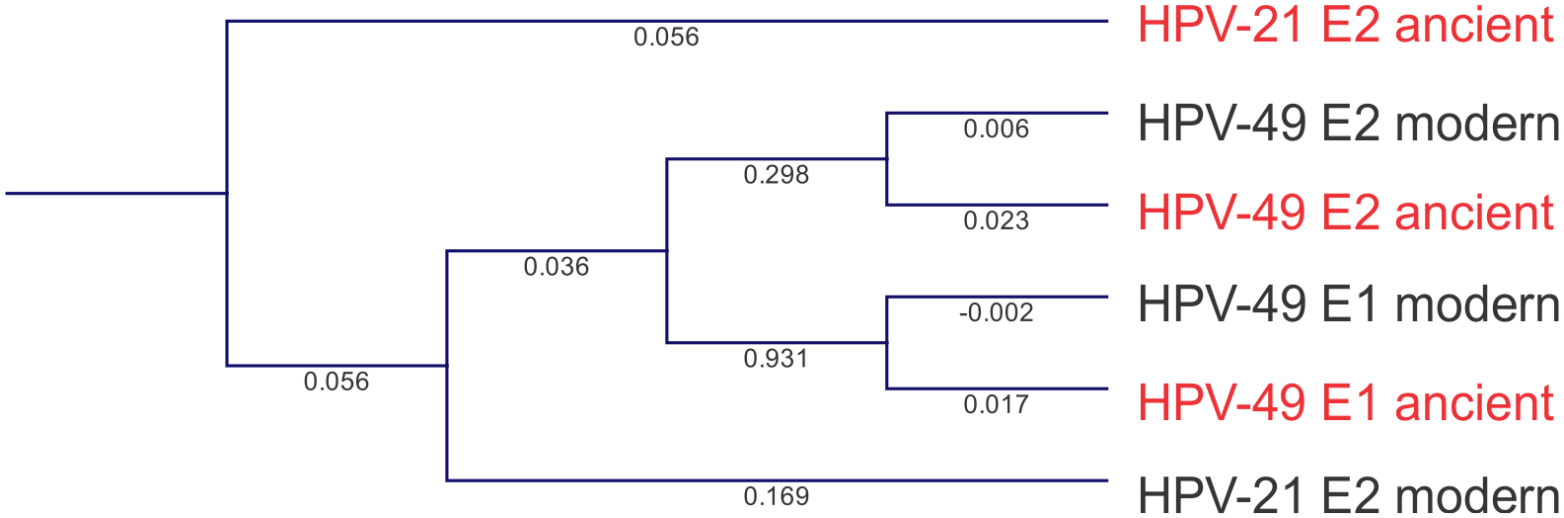
Phylogenetic reconstruction of ancient and extant HPV types 21 and 49 E1 and/or E2. Analyses were performed using maximum likelihood phylogeny using Neighbor Joining, and Jukes Cantor distance with bootstrap resampling (100 replicates). Branch lengths are shown.

While poor mapping was obtained with the phage database, sequences homologous to HPVs were identified in the mummified gut tissue and included HPV types 21 and 49 early gene 1 (E1) and/or early gene 2 (E2). Mapped sequences were BLASTn against the NCBInr database, confirming that the sequences were the best hit. Presumptive HPV-21 E2 from the mummy had its modern counterpart as the best hit, with a 100% identity (e-value = 2e^-115^). Presumptive HPV-49 E1and E2 from the mummy also had its modern counterpart as the best hit, with a 99 and 98% identity, respectively (e-value = 0.0) (S10 Dataset). We reconstructed the phylogeny of HPV types 21 and 49 and found that, with the exception of HPV type 2, all other HPV types were very similar to extant HPVs (Fig 9).

### Antibiotic-resistance

**Fig 10:**
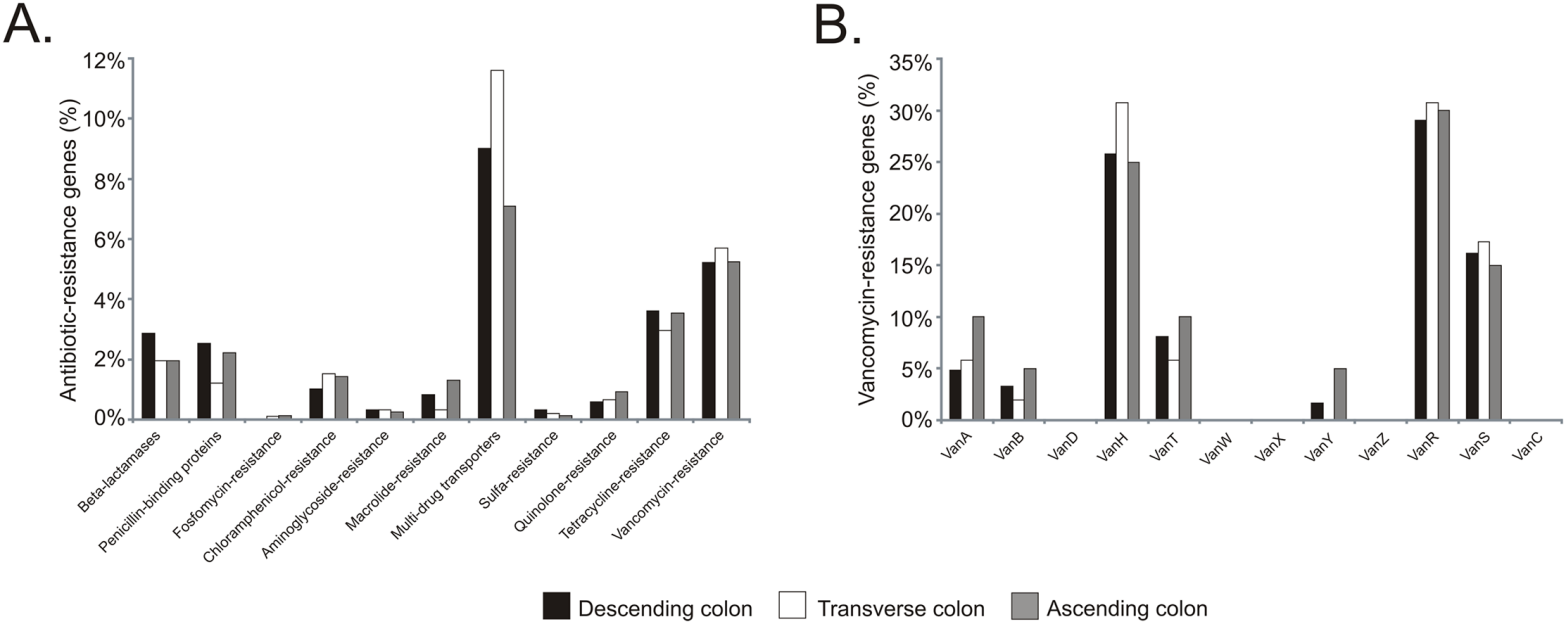
Percentage of putative antibiotic-resistance genes in mummified gut. Panel A shows the percentage of hits to genes associated with beta-lactamases, penicillin-binding proteins, resistance to fosfomycin, chloramphenicol, aminoglycosides, macrolides, sulfa, quinolones, tetracycline and vancomycin, and multi-drug transporters. Panel B shows the percentage of vancomycin genes including *vanA*, *vanB*, *vanH*, *vanT*, *vanY*, *vanR* and *vanS*.

Metagenomes functional categories showed that sequences associated with metabolism of aromatic compounds, nitrogen, sulfur, phosphorus, potassium, DNA, RNA and proteins, mobile genetic elements (phages, prophages and transposable elements), and virulence, disease and defense were represented in the mummified gut tissue (S2 Fig). We focused on antibiotic-resistance genes as part of the virulence, disease and defense category given that the mummy represents a unique opportunity to study antibiotic-resistance prior the antibiotic era. We performed a BLASTx analysis against CARDs to identify sequences that could belong to this category. Sequences homologous to antibiotic-resistance genes were identified in the descending (1,185 contigs), transverse (913 contigs) and ascending (762 contigs) colon. We found sequences associated with putative beta-lactamases, penicillin-binding proteins, resistance to fosfomycin, chloramphenicol, aminoglycosides, macrolides, sulfa, quinolones, tetracycline and vancomycin, and multi-drug transporters in the descending, transverse and ascending colon (Fig 10A). Results for the descending (S11 Dataset), transverse (S12 Dataset) and ascending colon (S13 Dataset) are included. With the emergence of vancomycin-resistance bacteria, and given that vancomycin-resistance has been more associated with the increased use of this antibiotic, we further investigated the percentage of putative vancomycin-resistance genes. Results show the presence of sequences sharing homology to *vanA*, *vanB*, *vanH*, *vanT*, *vanY*, *vanR* and *vanS* in the mummified gut specimens (Fig 10B).

## Discussion

One of the characteristics of authentic ancient DNA is its large level of fragmentation, consistent with the DNA isolated from the pre-Columbian Andean mummy in the present study [17, 32, 33]. Importantly, that the majority of the reads in the descending, transverse and ascending colon did not match any of the human gut, saliva, skin, and/or soil microbiomes suggests that no external sources of contamination influenced the findings reported in the present study. Results also suggest that handling of the mummy while being in the museum did not affect results, as demonstrated by the mummy’s sequences not matching to any of the skin microbiomes. Given that the majority of the reads did not match any of the human or soil microbiomes included in the SourceTracker analyses is also consistent with the study by Tito et al., 2012 reporting the gut microbiome of mummies [9]. In the case of the paleofeces, given that these were collected directly from the colon during the autopsy and the outermost portion was discarded prior analyses, it is difficult to exclude that the sequences matching soil microbiomes may be associated with dietary habits that included food contaminated with soil.

Numerous studies have demonstrated that a healthy human gut microbiome harbors bacterial species belonging to the Firmicutes, Bacteroidetes and Proteobacteria [34]. The natural mummification process may favor the preservation of Firmicutes, where the genus *Clostridium* is among the most abundant. These results are consistent with the study by Tito et al., where the vast majority of the coprolite’s microbiome from a South America mummy was mostly comprised by Firmicutes [9]. Results are also consistent with a previous study characterizing the gut microbiome during decomposition, where the Firmicutes comprised >95% of the gut microbiome prior the bloating stage [35], making Bacteroides and Proteobacteria relative abundances to decrease during the initial stages of decomposition. Also, while no studies have reported an association with an altered gut microbiome and Chaga’s disease, some studies have reported an increase in *Clostridium* spp. in association with other disease phenotypes such as Irritable Bowel Syndrome [36]; thus, it may be feasible that the high representation of *Clostridium* spp. in the mummified human gut may be a reflection of the pathological state (megavisceral syndrome) of the mummy. This needs to be studied with individuals afflicted with this parasite vis-à-vis the gut microbiome.

While we cannot rule out that differences in the microbiomes of the mummy and the coprolites samples may be due to sample collection and storage, these were sequenced using the same technology and analyzed in a similar manner. Therefore, it is feasible that the samples in fact possess differing microbiomes due to differences in the mechanisms of preservation (fossilization vs. mummification), the environment where these processes took place (i.e. coprolites were preserved in tropical soil [8], whereas the mummy was preserved in cold temperature and low oxygen levels), and/or differences in culture and health conditions, which often have an impact in the gut microbiome [13, 17]. Yet, given that we only are including one mummy from one culture, it is relatively difficult to make associations between the gut microbiome and cultural aspects (e.g. diet) as with the Saladoid and Huecoid cultures. In this previous study, we had access to more samples, which aid in the associations between the gut microbiome and dietary habits. In addition, we had archeological data supporting the 16S analyses. However, in the case of the pre-Columbian Andean mummy analyzed in the present study, we do not have any further archeological information or the 16S data of additional mummies to make associations between the gut microbiome and culture.

Interestingly, the paleofeces sample exhibited a different bacterial composition at the genus level when compared to the mummified colon specimens, and when compared to coprolites from the same era. The paleofeces had a higher representation of the genus *Turicibacter*, which was not detected in the pre-Columbian coprolites from the Saladoid and Huecoid cultures. *Turicibacter* spp. are non-spore formers, anaerobic, gram positive bacteria and little is known about its biology and ecology [37], but have been isolated from human feces [38]. *Turicibacter* spp. are also known to exhibit higher relative abundances after exposure to heavy metals [39]. The limited information available about the role of *Turicibacter* spp. as part of the human microbiome restricts our ability to suggest possible mechanisms by which these bacteria are highly abundant in the mummy’s paleofeces. However, it is feasible that the conditions encountered during natural mummification may favor its preservation. The present study warrants future studies about the role of *Turicibacter* during natural mummification and the processes that enable its preservation in the human gut.

While we were not able to identify sequences homologous to *C*. *botulinum* using 16S rRNA high-throughput sequencing, it may be due to its possible classification as *C*. *butyricum* (identified in the paleofeces and transverse colon), which often exhibits some sequence similarity with *C*. *botulinum* [40]. Presumptive *C*. *botulinum* in the mummy seemed to have diverged after *C*. *botulinum* type B strain 111. *C*. *botulinum* also has been identified in an European coprolite from the 14^th^ century [41], suggesting that this species have been prevalent in Europe and the Americas prior to European colonization. Also, the presence of sequences homologous to non-spore formers in the mummified human gut, including *Streptococcus* (paleofeces and descending colon), *Staphylococcus* (paleofeces), *Pseudomonas* (paleofeces, and descending, transverse and ascending colon) and *Bacillus* (paleofeces, descending and ascending colon) suggests that, similarly to amber formation, the natural mummification process may also favor the preservation of non-spore forming bacteria, opening the opportunity to elucidate possible mechanisms of survival in mummified gut tissue [14, 16, 42].

Notably, the gut microbiome of the pre-Columbian Andean mummy was not restricted to bacterial communities as predicated by the metagenome analyses showing microbial eukaryotic sequences. Reads sharing homology to modern *T*. *cruzi* and the phylogenetic analyses support previous studies indicating the presence of Chagas’ disease in the Americas prior to Spanish colonization. Results also suggest that metagenomics may augment results obtained from paleopathological analyses. While sequences associated with presumptive *T*. *cruzi* were identified, our data were not sufficient to reconstruct the complete genome of the ancient *T*. *cruzi*, which may better provide information about the evolution of this pathogen. From the phylogenetic analysis, it is evident that sequences homologous to *T*. *cruzi* detected in the mummy have a more remote origin compared to modern *T*. *cruzi* strains. The presence of sequences homologous to pathogenic eukaryotes, such as *T*. *cruzi*, in the ancient human gut may also suggest that an integrated approach using next-generation sequencing and paleopathological analyses may better demonstrate if the presence of a pathogen may be a feasible cause of disease and possible demise.

Few studies have reported the presence of viral communities in ancient human specimens. A previous study reported sequences associated with siphoviruses in a 14^th^ century European coprolite [30]. That we were not able to identify phage sequences indicates that our extraction method was not sensitive enough for the detection of phages. Unlike viral metagenomics that may employ CsCl gradients for the recovery of viruses, whole-sample sequencing may not sufficiently capture all the possible viral communities in a sample. This previous study, however, did not identify a great proportion of eukaryotic viruses, possibly due to the sequencing of the most common CsCl fraction, which mostly recovers phages. The presence of sequences homologous to HPVs in the mummified gut (descending colon) was particularly surprising as very few studies have reported HPV sequences in human mummies [43]. A previous study did report a perianal condyloma caused by HPV type 18 and JC9813 in Mary of Aragon (16^th^ century). The present study suggests that metagenomics could be used to identify ancient pathogenic viruses that often do not show any abnormalities [44]. While we did not detect sequences homologous to high-risk HPV sequences or have paleopathological data indicating an HPV infection, HPV-21 has been associated with epidermodysplasia verruciformis, a genetic hereditary skin disorder associated with a high risk of carcinoma of the skin [45], and HPV-49 has been identified in flat warts [46]. The detection of HPV sequences in an 11^th^ century A.D. pre-Columbian Andean mummy provides evidence that HPVs also were prevalent in different geographical regions. Notably, the majority of sequences homologous to HPVs in the mummy were very similar to modern counterparts, suggesting that the virus may have a slow evolution. Our data also support a previous study suggesting that HPVs may have been introduced to the Americas through ancient human migrations rather than European colonization [47].

Similarly, the origin of antibiotic-resistance has also been questioned. Antibiotic-resistance genes have been found in 30,000 year-old sediments, indicating that antibiotic-resistance is not only ancient, but also originates from the environment [12, 48]. Antibiotics are widely used in modern societies, resulting in the emergence of microbes that harbor antibiotic-resistance genes that confer this resistance. However, putative antibiotic-resistance genes also have been detected in ancient human specimens including the oral cavity [11], and in bacteriophages from a 14^th^ century European coprolite [30]. A recent study also identified antibiotic-resistance genes in isolated indigenous cultures from South America [49]. While this culture has remained largely unaltered for the last 11,000 years, there has been some contact with westernized cultures; thus, it is feasible that antibiotic-resistance genes may have been acquired through their transmission from westernized subjects. The presence of antibiotic-resistance genes in an 11^th^ century pre-Columbian Andean mummy is intriguing as antibiotics were introduced recently. The presence of beta-lactam antibiotic resistance is certainly not unexpected in any culture, as would be in the case of resistance to any natural rather than a semi- or completely synthetic antibiotic as a result of exposure to natural antibiotic-producing microbiota originating from the environment (e.g. soil); however, vancomycin, particularly, was discovered more than 50 years ago, and vancomycin-resistance genes have been mainly implicated with the increased use of this antibiotic [50]. The presence of antibiotic-resistance genes in the ancient human gut microbiome clearly indicates that these genes pre-date therapeutical use of these compounds and that they are not necessarily associated to a selective pressure of antibiotics use.

## Conclusions

The natural mummification process of the human gut results in the preservation of bacterial DNA, with the Firmicutes being among the most abundant. The mummy’s paleofeces exhibited a different microbiome profile when compared to coprolites, where *Turicibacter* was the most abundant genus. *Streptococcus*, *Staphylococcus*, *Bacillus* and *Pseudomonas* sequences were also identified in the mummified gut, opening the opportunity to investigate possible mechanisms by which these bacteria are preserved. The detection of sequences homologous to those of pathogens such as *T*. *cruzi* and HPV indicate their presence in the Americas prior to European colonization. Identification of putative antibiotic-resistance genes indicates their pre-existence in the human gut prior the introduction of antibiotics. Identification of pathogens and antibiotic-resistance genes in ancient human specimens will aid in the understanding of the evolution of pathogens as a way to treat and prevent diseases caused by bacteria, microbial eukaryotes and viruses.

While museum specimens are an invaluable source of information and can be used to study the evolution of infectious diseases [51], one of the limitations of such studies is that further analyses are needed to confirm that no external sources of contamination contributed to the findings. Also, given that authentic ancient DNA yields very small fragments may limit our ability to map or identify sequences sharing homology to large genomes, as those from eukaryotic microorganisms. Future studies are also needed to understand the metabolic pathways during the natural mummification process and how these differ from other processes such as fossilization of fecal material. Data presented here also warrant future studies on how the gut microbiome of ancient samples is altered by taphonomic processes.

## Supporting Information

S1 Dataset *Clostridium botulinum* sequences for phylogenetic analyses. (FA)

S2 Dataset Descending colon *Trypanosoma cruzi* CL Brener BLASTn results. (TXT)

S3 Dataset Descending colon *Trypanosoma cruzi* Dm28c BLASTn results. (TXT)

S4 Dataset Descending colon *Trypanosoma cruzi* Esmeraldo BLASTn results. (TXT)

S5 Dataset Descending colon *Trypanosoma cruzi* JR cl4 BLASTn results. (TXT)

S6 Dataset Descending colon *Trypanosoma cruzi* Marinkellei BLASTn results. (TXT)

S7 Dataset Descending colon *Trypanosoma cruzi* Sylvio BLASTn results. (TXT)

S8 Dataset Descending colon *Trypanosoma cruzi* Tula cl2 BLASTn results. (TXT)

S9 Dataset *Trypanosoma cruzi* large ribosomal subunit alpha sequences phylogenetic analyses. (FA)

S10 Dataset HPV early genes 1and/or 2 sequences for phylogenetic analyses. (FA)

S11 Dataset Descending colon CARDs BLASTx results. (TXT)

S12 Dataset Transverse colon_CARDs BLASTx results. (TXT)

S13 Dataset Ascending colon_CARDs BLASTx results. (TXT)

S1 Fig Alphararefaction curves showing chao1 values of bacterial diversity by 16S rRNA gene sequences in paleofeces, and descending, transverse and ascending colon. (TIF)

S2 Fig Bar charts of functional categories relative abundances (%) of mummified descending (black), transverse (white) and ascending colon (gray). (TIF)

S1 File Samples used for the procrustes and SourceTracker analyses. (TXT)

S1 Table Identification at genus level of the 16S rRNA sequences generated by highthroughput sequencing of the Pre Columbian Andean mummy's specimens. (DOCX)

S2 Table Presumptive bacterial species in mummy’s paleofeces. (DOCX)

S3 Table Presumptive bacterial species in mummy’s descending colon. (DOCX)

S4 Table Presumptive bacterial species in mummy’s transverse colon. (DOCX)

S5 Table Presumptive bacterial species in mummy’s ascending colon. (DOCX)

S6 Table BLASTn analyses of mummy’s microbiome (descending colon) against available *Trypanosoma cruzi* strains. (DOCX)
