# Supplementary figures and images for: Gut Microbiome of an 11th Century A.D. Pre-Columbian Andean Mummy
Source: PLoS One. 2015 Sep 30;10(9):e0138135. doi: 10.1371/journal.pone.0138135 (PMC4589460; doi:10.1371/journal.pone.0138135)

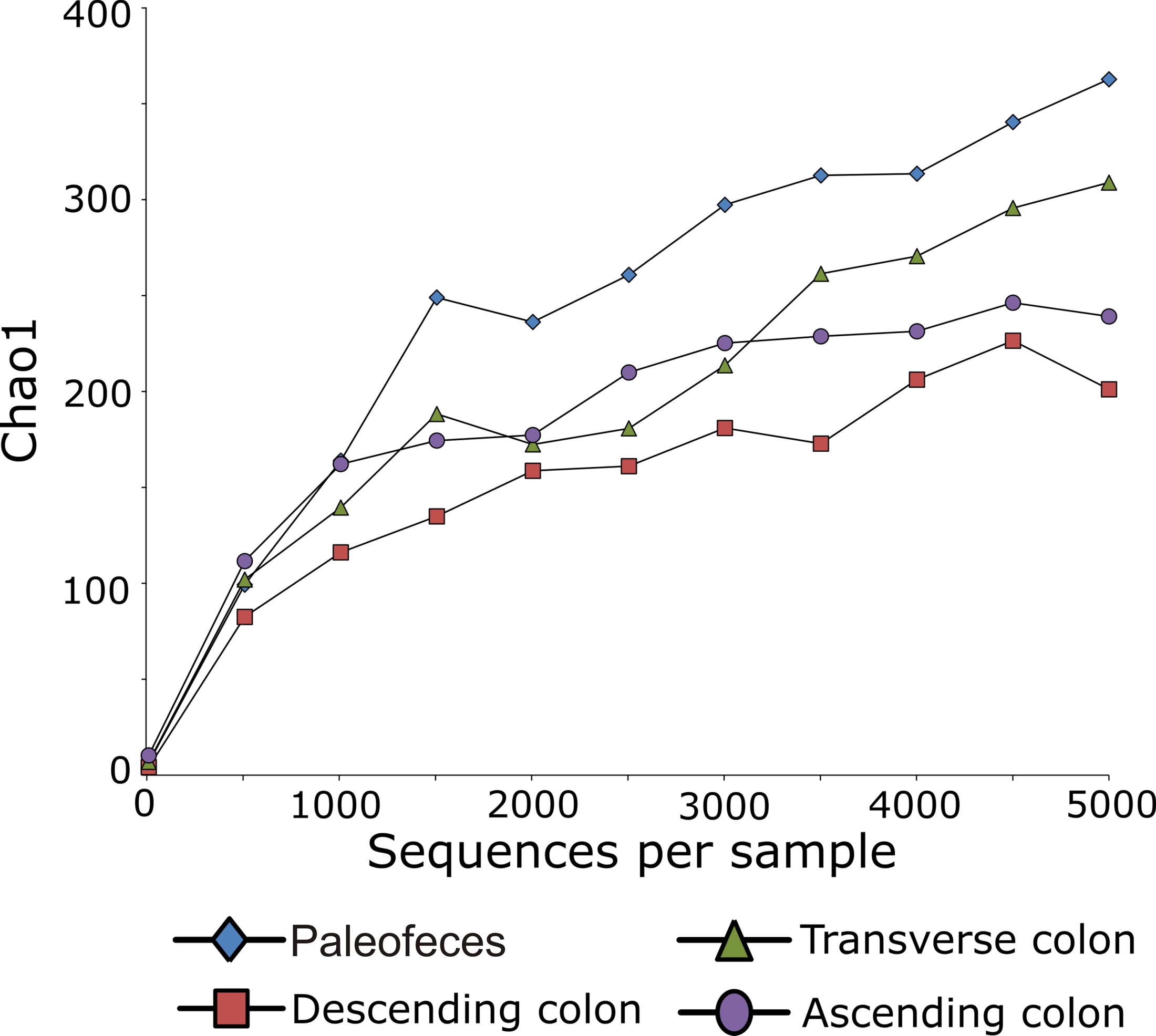

Supplement: S1 Fig — (TIF) [file pone.0138135.s014.tif]

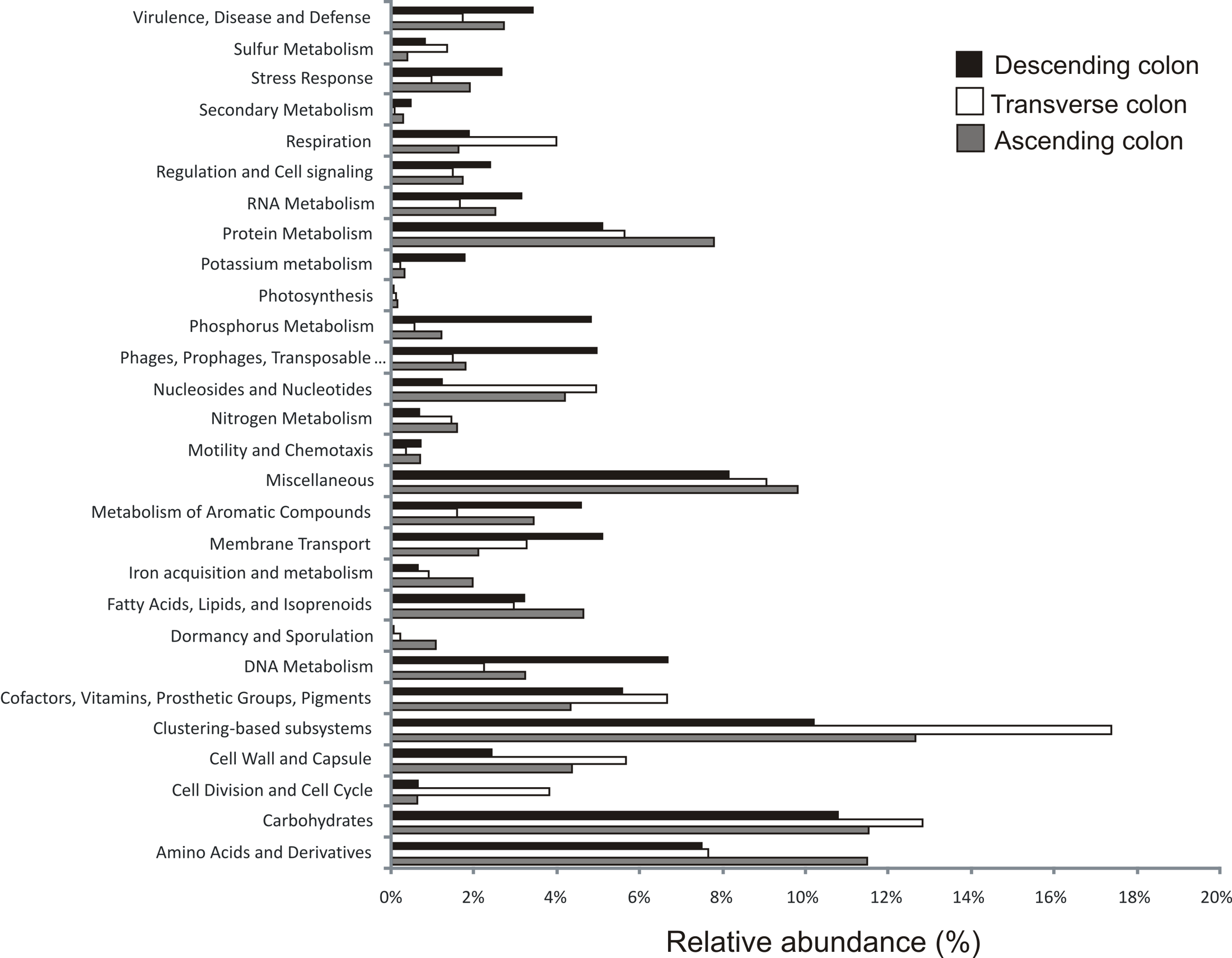

Supplement: S2 Fig — (TIF) [file pone.0138135.s015.tif]
