# Supplementary material for: Gut Microbiome of an 11th Century A.D. Pre-Columbian Andean Mummy
Source: PLoS One. 2015 Sep 30;10(9):e0138135. doi: 10.1371/journal.pone.0138135 (PMC4589460; doi:10.1371/journal.pone.0138135)
Supplement: S1 Table — (DOCX) [file pone.0138135.s017.docx]

**Supplemental Table 1.** Identification at genus level of the 16S rRNA sequences generated by highthroughput sequencing of the Pre Columbian Andean mummy's specimens.

| **Taxon** | **Paleofeces** | **Descending colon** | **Transverse colon** | **Ascending colon** |
| --- | --- | --- | --- | --- |
| *Clostridium* | 7.7% | 81.4% | 96.2% | 68.3% |
| *Turicibacter* | 89.2% | 5.1% | 2.7% | 19.8% |
| *Cryocola* | 0.0% | 3.4% | 0.0% | 0.7% |
| *Sphingomonas* | 0.0% | 2.1% | 0.1% | 0.2% |
| *Pseudomonas* | 0.2% | 1.5% | 0.1% | 0.5% |
| *Aggregatibacter* | 0.0% | 0.8% | 0.0% | 0.0% |
| *Streptococcus* | 0.1% | 0.8% | 0.0% | 0.2% |
| *Bacillus* | 0.1% | 0.5% | 0.1% | 1.1% |
| *Virgibacillus* | 0.1% | 0.5% | 0.0% | 1.5% |
| *Corynebacterium* | 0.0% | 0.4% | 0.0% | 0.0% |
| *Tepidimicrobium* | 0.0% | 0.4% | 0.1% | 0.3% |
| *Veillonella* | 0.0% | 0.4% | 0.0% | 0.0% |
| *Paenibacillus* | 0.0% | 0.2% | 0.1% | 3.7% |
| *Acinetobacter* | 0.0% | 0.2% | 0.1% | 0.1% |
| *Methylobacterium* | 0.0% | 0.2% | 0.0% | 0.2% |
| *Neisseria* | 0.0% | 0.2% | 0.0% | 0.0% |
| *Sarcina* | 2.1% | 0.2% | 0.2% | 0.2% |
| *Paenisporosarcina* | 0.0% | 0.2% | 0.0% | 0.7% |
| *Ammoniphilus* | 0.0% | 0.2% | 0.0% | 0.0% |
| *Thermoanaerobacterium* | 0.0% | 0.2% | 0.0% | 0.0% |
| *Prevotella* | 0.0% | 0.1% | 0.0% | 0.0% |
| *Rhodoplanes* | 0.0% | 0.1% | 0.0% | 0.0% |
| *Alkaliphilus* | 0.0% | 0.1% | 0.0% | 0.0% |
| *Brachybacterium* | 0.0% | 0.1% | 0.0% | 0.0% |
| *Brevibacterium* | 0.0% | 0.1% | 0.0% | 0.0% |
| *Paracoccus* | 0.0% | 0.1% | 0.0% | 0.0% |
| *Saccharopolyspora* | 0.0% | 0.1% | 0.0% | 0.0% |
| *Sporanaerobacter* | 0.0% | 0.1% | 0.0% | 0.5% |
| *Ruminococcus* | 0.1% | 0.0% | 0.0% | 0.0% |
| *Fusobacterium* | 0.0% | 0.0% | 0.0% | 0.1% |
| *Cohnella* | 0.0% | 0.0% | 0.0% | 0.1% |
| *Comamonas* | 0.0% | 0.0% | 0.0% | 0.2% |
| *Rubellimicrobium* | 0.0% | 0.0% | 0.0% | 0.2% |
| *Shewanella* | 0.0% | 0.0% | 0.0% | 0.1% |
| *Sporosarcina* | 0.0% | 0.0% | 0.0% | 1.4% |
