# Supplementary material for: Gut Microbiome of an 11th Century A.D. Pre-Columbian Andean Mummy
Source: PLoS One. 2015 Sep 30;10(9):e0138135. doi: 10.1371/journal.pone.0138135 (PMC4589460; doi:10.1371/journal.pone.0138135)
Supplement: S2 Table — (DOCX) [file pone.0138135.s018.docx]

**Supplemental Table 2.** Presumptive bacterial species in mummy’s paleofeces.

| **Description** | **Identity (%)** | **E-value** | **Accession** |
| --- | --- | --- | --- |
| *Clostridium aldenense* | 95.24 | 1.32E-121 | NR_043680 |
| *Clostridium bifermentans* | 94.63 | 4.02E-102 | NR_113323 |
| *Clostridium cellobioparum* | 92.67 | 2.24E-112 | NR_113360 |
| *Clostridium cellulolyticum* | 93.04 | 1.61E-101 | NR_102768 |
| *Clostridium colinum* | 98.53 | 4.31E-134 | NR_026151 |
| *Clostridium jejuense* | 89.74 | 1.61E-101 | NR_025796 |
| *Clostridium saccharogumia* | 92.65 | 9.43E-111 | NR_043550 |
| *Clostridium sordellii* | 99.63 | 2.37E-137 | NR_113140 |
| *Acetitomaculum ruminis* | 93.41 | 4.30E-115 | NR_044713 |
| *Actinomyces odontolyticus* | 98.17 | 1.83E-132 | NR_114395 |
| *Alicyclobacillus contaminans* | 86.45 | 4.93E-89 | NR_114203 |
| *Anaerobacterium chartisolvens* | 89.01 | 1.01E-97 | NR_125464 |
| *Anaerotruncus colihominis* | 91.2 | 2.97E-91 | NR_027558 |
| *Bacillus circulans* | 98.17 | 1.83E-132 | NR_118445 |
| *Bacillus hemicentroti* | 92.74 | 2.15E-99 | NR_109010 |
| *Bacillus toyonensis* | 99.62 | 5.27E-133 | NR_121761 |
| *Bacteroides stercorirosoris* | 85.77 | 3.76E-84 | NR_113207 |
| *Brucella ceti* | 98.18 | 6.32E-132 | NR_121762 |
| *Caloramator proteoclasticus* | 87.6 | 6.00E-88 | NR_026265 |
| *Cerasibacillus quisquiliarum* | 98.9 | 3.54E-135 | NR_114087 |
| *Cloacibacterium rupense* | 99.27 | 8.32E-137 | NR_114274 |
| *Clostridium algidicarnis* | 97.18 | 5.24E-114 | NR_041746 |
| *Clostridium butyricum* | 95.99 | 8.32E-137 | NR_113244 |
| *Clostridium cavendishii* | 96 | 3.80E-122 | NR_115711 |
| *Clostridium chartatabidum* | 99.5 | 2.17E-98 | NR_029239 |
| *Clostridium chauvoei* | 98 | 1.36E-94 | NR_026013 |
| *Clostridium difficile* | 95.24 | 9.38E-130 | NR_074454 |
| *Clostridium disporicum* | 95.09 | 8.32E-137 | NR_026491 |
| *Clostridium putrefaciens* | 94.58 | 7.42E-125 | NR_113324 |
| *Clostridium quinii* | 93.44 | 2.51E-71 | NR_026149 |
| *Clostridium sartagoforme* | 93.41 | 4.92E-127 | NR_026490 |
| *Clostridium tertium* | 93.05 | 3.03E-83 | NR_113325 |
| *Coprococcus eutactus* | 95.97 | 2.55E-124 | NR_044049 |
| *Eubacterium coprostanoligenes* | 93.59 | 8.93E-98 | NR_104907 |
| *Eubacterium moniliforme* | 92.92 | 5.25E-81 | NR_104892 |
| *Eubacterium ruminantium* | 88.64 | 3.55E-97 | NR_024661 |
| *Eubacterium siraeum* | 95.97 | 2.55E-124 | NR_102935 |
| *Gemmatimonas aurantiaca* | 85.82 | 3.80E-84 | NR_074708 |
| *Intestinibacter bartlettii* | 99.26 | 5.35E-100 | NR_027573 |
| *Lactobacillus leichmannii* | 98.91 | 4.35E-134 | NR_104969 |
| *Lutispora thermophila* | 90.15 | 4.63E-102 | NR_041236 |
| *Natronincola histidinovorans* | 94.79 | 2.88E-77 | NR_026455 |
| *Oceanobacillus polygoni* | 97.44 | 9.50E-130 | NR_114348 |
| *Paenibacillus ginsengarvi* | 94.49 | 2.38E-118 | NR_041519 |
| *Pseudomonas lini* | 100 | 1.32E-121 | NR_029042 |
| *Romboutsia ilealis* | 97.08 | 4.90E-127 | NR_125597 |
| *Ruminococcus bromii* | 95.62 | 1.08E-122 | NR_025930 |
| *Ruminococcus champanellensis* | 95.62 | 9.50E-130 | NR_102884 |
| *Sarcina maxima* | 91.39 | 7.93E-99 | NR_026147 |
| *Sarcina ventriculi* | 98.9 | 4.31E-134 | NR_026146 |
| *Spiroplasma apis* | 83.15 | 1.50E-76 | NR_121708 |
| *Spiroplasma velocicrescens* | 87.45 | 4.79E-89 | NR_025713 |
| *Sporobacter termitidis* | 94.51 | 6.84E-119 | NR_044972 |
| *Staphylococcus agnetis* | 96.72 | 1.72E-126 | NR_117863 |
| *Staphylococcus pasteuri* | 97.07 | 1.16E-128 | NR_121749 |
| *Streptococcus parasanguinis* | 98.16 | 6.37E-132 | NR_074109 |
| *Terrisporobacter mayombei* | 98.02 | 6.62E-119 | NR_104744 |
| *Thermoflavimicrobium dichotomicum* | 92.67 | 2.71E-111 | NR_025005 |
| *Tissierella praeacuta* | 92.65 | 9.43E-111 | NR_044860 |
| *Turicibacter sanguinis* | 92.65 | 3.13E-83 | NR_028816 |
| *Vallitalea pronyensis* | 88.64 | 3.55E-97 | NR_125677 |
