# Supplementary material for: Gut Microbiome of an 11th Century A.D. Pre-Columbian Andean Mummy
Source: PLoS One. 2015 Sep 30;10(9):e0138135. doi: 10.1371/journal.pone.0138135 (PMC4589460; doi:10.1371/journal.pone.0138135)
Supplement: S3 Table — (DOCX) [file pone.0138135.s019.docx]

**Supplemental Table 3.** Presumptive bacterial species in mummy’s descending colon.

| **Description** | **Identity (%)** | **E-value** | **Accession** |
| --- | --- | --- | --- |
| *Abiotrophia defectiva* | 99.63 | 6.83E-138 | NR_025863 |
| *Acinetobacter johnsonii* | 99.27 | 8.32E-137 | NR_117624 |
| *Aggregatibacter aphrophilus* | 93.07 | 7.80E-112 | NR_102838 |
| *Alkaliphilus halophilus* | 93.38 | 2.21E-112 | NR_116395 |
| *Alloprevotella rava* | 87.91 | 2.70E-92 | NR_118334 |
| *Anaerobacterium chartisolvens* | 87.75 | 5.67E-68 | NR_125464 |
| *Anaerostipes hadrus* | 87.55 | 2.24E-93 | NR_104799 |
| *Anoxybacillus flavithermus* | 99.01 | 2.61E-98 | NR_074667 |
| *Arthrobacter phenanthrenivorans* | 99.27 | 8.32E-137 | NR_074770 |
| *Atopostipes suicloacalis* | 98.9 | 3.54E-135 | NR_028835 |
| *Aureimonas ferruginea* | 99.27 | 8.32E-137 | NR_118423 |
| *Bacillus idriensis* | 98.53 | 4.31E-134 | NR_043268 |
| *Bacillus luteolus* | 96.34 | 5.99E-126 | NR_108638 |
| *Bacillus salsus* | 97.45 | 1.42E-127 | NR_109135 |
| *Bacteroides rodentium* | 93.07 | 6.43E-113 | NR_113072 |
| *Blastococcus aggregatus* | 99.27 | 8.32E-137 | NR_114864 |
| *Brachybacterium paraconglomeratum* | 98.9 | 3.54E-135 | NR_113401 |
| *Bradyrhizobium japonicum* | 96.7 | 4.88E-108 | NR_119191 |
| *Brevibacterium oceani* | 99.27 | 2.92E-136 | NR_042458 |
| *Capnocytophaga sputigena* | 99.63 | 6.83E-138 | NR_113564 |
| *Caulobacter segnis* | 98.9 | 4.29E-134 | NR_074208 |
| *Clostridium bifermentans* | 95.24 | 1.95E-119 | NR_113323 |
| *Clostridium chromiireducens* | 95.24 | 1.11E-82 | NR_122090 |
| *Clostridium disporicum* | 99.6 | 8.39E-124 | NR_026491 |
| *Clostridium indolis* | 93.41 | 6.35E-113 | NR_026493 |
| *Clostridium methylpentosum* | 91.21 | 7.28E-106 | NR_029355 |
| *Clostridium peptidivorans* | 88.69 | 1.24E-96 | NR_025019 |
| *Clostridium putrefaciens* | 97.78 | 1.76E-106 | NR_113324 |
| *Clostridium saccharobutylicum* | 93.77 | 5.21E-114 | NR_122061 |
| *Clostridium sporogenes* | 99.27 | 8.32E-137 | NR_118630 |
| *Clostridium tetani* | 97.98 | 7.34E-72 | NR_074498 |
| *Clostridium uliginosum* | 97.99 | 4.28E-115 | NR_028920 |
| *Conexibacter woesei* | 90.11 | 1.60E-101 | NR_074830 |
| *Corynebacterium terpenotabidum* | 99.27 | 2.39E-137 | NR_121699 |
| *Flavonifractor plautii* | 95.97 | 2.55E-124 | NR_043142 |
| *Geobacillus stearothermophilus* | 98.17 | 1.83E-132 | NR_116987 |
| *Intestinibacter bartlettii* | 95.96 | 1.07E-122 | NR_027573 |
| *Leifsonia xyli* | 99.27 | 8.32E-137 | NR_074130 |
| *Lewinella marina* | 91.58 | 5.97E-107 | NR_114170 |
| *Methylobacterium jeotgali* | 99.63 | 6.83E-138 | NR_043878 |
| *Neisseria cinerea* | 99.63 | 6.83E-138 | NR_121687 |
| *Oceanobacillus luteolus* | 97.07 | 1.16E-128 | NR_126270 |
| *Oscillibacter ruminantium* | 92.34 | 4.04E-109 | NR_118156 |
| *Oxalophagus oxalicus* | 97.8 | 2.23E-131 | NR_036979 |
| *Polaromonas sp. JS666* | 98.18 | 7.80E-131 | NR_074725 |
| *Prevotella melaninogenica* | 98.53 | 4.31E-134 | NR_102895 |
| *Pseudomonas lini* | 96.5 | 1.08E-122 | NR_029042 |
| *Rhodobacter sphaeroides* | 96.34 | 5.99E-126 | NR_074171 |
| *Rhodoplanes roseus* | 96.35 | 2.10E-125 | NR_115515 |
| *Romboutsia ilealis* | 96.09 | 1.42E-114 | NR_125597 |
| *Romboutsia lituseburensis* | 95.24 | 1.31E-121 | NR_118728 |
| *Saccharopolyspora phatthalungensis* | 96.7 | 4.92E-127 | NR_117349 |
| *Salirhabdus euzebyi* | 96.34 | 6.25E-99 | NR_042538 |
| *Sphingomonas echinoides* | 96.24 | 8.32E-137 | NR_113806 |
| *Streptococcus dentisani* | 99.26 | 2.89E-136 | NR_117719 |
| *Streptococcus thermophilus* | 99.63 | 2.37E-137 | NR_118998 |
| *Terrisporobacter mayombei* | 98.53 | 2.29E-118 | NR_104744 |
| *Thermoanaerobacterium xylanolyticum* | 99.27 | 8.32E-137 | NR_102771 |
| *Tissierella creatinophila* | 94.87 | 1.96E-119 | NR_037028 |
| *Tissierella praeacuta* | 93.75 | 1.23E-115 | NR_044860 |
| *Vampirovibrio chlorellavorus* | 85.71 | 8.84E-86 | NR_104911 |
| *Veillonella denticariosi* | 95.96 | 2.54E-124 | NR_044153 |
| *Veillonella parvula* | 99.26 | 2.89E-136 | NR_074980 |
| *Virgibacillus siamensis* | 98.9 | 3.54E-135 | NR_112738 |
