# Supplementary material for: Gut Microbiome of an 11th Century A.D. Pre-Columbian Andean Mummy
Source: PLoS One. 2015 Sep 30;10(9):e0138135. doi: 10.1371/journal.pone.0138135 (PMC4589460; doi:10.1371/journal.pone.0138135)
Supplement: S4 Table — (DOCX) [file pone.0138135.s020.docx]

**Supplemental Table 4.** Presumptive bacterial species in mummy’s transverse colon.

| **Description** | **Identity (%)** | **E-value** | **Accession** |
| --- | --- | --- | --- |
| *Acinetobacter johnsonii* | 93.04 | 1.83E-113 | NR_117624 |
| *Acinetobacter lwoffii* | 99.27 | 8.32E-137 | NR_113346 |
| *Anaerobacterium chartisolvens* | 87.59 | 9.51E-92 | NR_125464 |
| *Anaerosalibacter bizertensis* | 91.51 | 5.97E-107 | NR_117999 |
| *Azospira restricta* | 97.45 | 4.04E-128 | NR_044023 |
| *Bacillus salsus* | 99.27 | 8.32E-137 | NR_109135 |
| *Bacteroides xylanolyticus* | 94.85 | 5.59E-120 | NR_104899 |
| *Clostridium aldenense* | 94.89 | 5.64E-120 | NR_043680 |
| *Clostridium algidicarnis* | 94.51 | 1.62E-120 | NR_041746 |
| *Clostridium butyricum* | 98.18 | 2.73E-130 | NR_113244 |
| *Clostridium cavendishii* | 93.77 | 3.54E-116 | NR_115711 |
| *Clostridium chromiireducens* | 93.4 | 2.41E-85 | NR_122090 |
| *Clostridium cylindrosporum* | 88.64 | 1.73E-88 | NR_026492 |
| *Clostridium disporicum* | 98.18 | 5.30E-108 | NR_026491 |
| *Clostridium indolis* | 94.87 | 1.95E-119 | NR_026493 |
| *Clostridium methylpentosum* | 88.36 | 6.41E-94 | NR_029355 |
| *Clostridium puniceum* | 94.91 | 2.40E-118 | NR_119031 |
| *Clostridium putrefaciens* | 94.7 | 1.08E-122 | NR_113324 |
| *Clostridium sardiniense* | 95.26 | 4.63E-121 | NR_112226 |
| *Clostridium septicum* | 96.69 | 1.71E-126 | NR_026020 |
| *Clostridium tertium* | 96.34 | 1.32E-121 | NR_113325 |
| *Clostridium tetani* | 95.97 | 1.07E-122 | NR_074498 |
| *Clostridium uliginosum* | 95.72 | 1.81E-113 | NR_028920 |
| *Corynebacterium coyleae* | 98.18 | 6.40E-132 | NR_044905 |
| *Corynebacterium tuberculostearicum* | 99.27 | 2.39E-137 | NR_028975 |
| *Empedobacter brevis* | 94.51 | 6.84E-119 | NR_042471 |
| *Flavobacterium subsaxonicum* | 99.27 | 1.01E-135 | NR_115085 |
| *Gluconacetobacter liquefaciens* | 95.24 | 1.32E-121 | NR_113406 |
| *Halolactibacillus halophilus* | 92.31 | 9.50E-111 | NR_113954 |
| *Halothiobacillus kellyi* | 82.02 | 3.56E-58 | NR_025030 |
| *Hespellia stercorisuis* | 87.18 | 1.15E-90 | NR_025207 |
| *Intestinibacter bartlettii* | 95.59 | 1.08E-122 | NR_027573 |
| *Lentibacillus salis* | 92.73 | 3.33E-110 | NR_043170 |
| *Ornithinibacillus scapharcae* | 94.53 | 2.40E-118 | NR_117927 |
| *Paenibacillus cellulositrophicus* | 95.59 | 1.08E-122 | NR_116564 |
| *Paenibacillus taiwanensis* | 92.78 | 6.00E-107 | NR_044007 |
| *Prevotella loescheii* | 99.25 | 5.25E-133 | NR_113109 |
| *Pseudomonas lini* | 94.89 | 8.30E-118 | NR_029042 |
| *Romboutsia ilealis* | 94.55 | 1.17E-109 | NR_125597 |
| *Ruminiclostridium thermocellum* | 92.31 | 9.50E-111 | NR_074629 |
| *Sphingobium xenophagum* | 99.27 | 8.32E-137 | NR_114554 |
| *Sphingomonas ginsenosidivorax* | 98.58 | 1.78E-99 | NR_117830 |
| *Sporanaerobacter acetigenes* | 90.46 | 1.80E-87 | NR_117381 |
| *Sporosarcina globispora* | 98.91 | 1.50E-133 | NR_118912 |
| *Tepidiphilus margaritifer* | 98.9 | 4.29E-134 | NR_025556 |
| *Terrisporobacter mayombei* | 98.53 | 1.22E-95 | NR_104744 |
| *Tissierella creatinophila* | 96.69 | 1.46E-114 | NR_037028 |
| *Tissierella praeacuta* | 95.59 | 2.20E-112 | NR_044860 |
| *Turicibacter sanguinis* | 95.4 | 3.54E-116 | NR_028816 |
| *Ureibacillus thermosphaericus* | 98.9 | 3.54E-135 | NR_040961 |
| *Virgibacillus carmonensis* | 93.8 | 1.24E-115 | NR_025481 |
| *Virgibacillus halophilus* | 96.6 | 2.39E-65 | NR_041358 |
