# Supplementary material for: Gut Microbiome of an 11th Century A.D. Pre-Columbian Andean Mummy
Source: PLoS One. 2015 Sep 30;10(9):e0138135. doi: 10.1371/journal.pone.0138135 (PMC4589460; doi:10.1371/journal.pone.0138135)
Supplement: S5 Table — (DOCX) [file pone.0138135.s021.docx]

**Supplemental Table 5.** Presumptive bacterial species in mummy’s ascending colon.

| **Description** | **Identity (%)** | **E-value** | **Accession** |
| --- | --- | --- | --- |
| *Anaerosalibacter bizertensis* | 99.26 | 2.89E-136 | NR_117999 |
| *Aquabacterium fontiphilum* | 99.62 | 5.29E-133 | NR_044322 |
| *Bacillus abyssalis* | 96.34 | 5.99E-126 | NR_109671 |
| *Bacillus oceanisediminis* | 97.44 | 9.50E-130 | NR_118440 |
| *Bacillus salsus* | 97.07 | 1.16E-128 | NR_109135 |
| *Bacillus thermoamylovorans* | 98.9 | 1.01E-135 | NR_117028 |
| *Caloramator proteoclasticus* | 89.01 | 8.34E-99 | NR_026265 |
| *Catabacter hongkongensis* | 90.88 | 2.55E-105 | NR_115269 |
| *Chungangia koreensis* | 98.9 | 3.54E-135 | NR_117554 |
| *Clostridium cochlearium* | 95.26 | 4.63E-121 | NR_113026 |
| *Clostridium disporicum* | 92.96 | 1.32E-121 | NR_026491 |
| *Clostridium estertheticum* | 98.54 | 1.51E-133 | NR_118995 |
| *Clostridium jejuense* | 88.35 | 3.36E-91 | NR_025796 |
| *Clostridium lavalense* | 94.87 | 1.61E-120 | NR_044289 |
| *Clostridium oceanicum* | 91.64 | 2.09E-106 | NR_117136 |
| *Clostridium putrefaciens* | 97.64 | 4.90E-127 | NR_113324 |
| *Clostridium tetani* | 97.49 | 1.01E-109 | NR_074498 |
| *Clostridium uliginosum* | 96.99 | 1.60E-120 | NR_028920 |
| *Cohnella lupini* | 99.62 | 1.84E-132 | NR_125663 |
| *Comamonas testosteroni* | 98.53 | 2.02E-92 | NR_102841 |
| *Desulfonema magnum* | 95.02 | 1.09E-84 | NR_025990 |
| *Escherichia fergusonii* | 99.27 | 1.01E-135 | NR_074902 |
| *Fusobacterium simiae* | 98.99 | 1.23E-134 | NR_113318 |
| *Hyphomicrobium hollandicum* | 96.46 | 3.80E-89 | NR_026428 |
| *Intestinibacter bartlettii* | 95.24 | 2.89E-136 | NR_027573 |
| *Jeotgalibacillus soli* | 99.27 | 8.32E-137 | NR_125726 |
| *Lautropia mirabilis* | 98.53 | 4.31E-134 | NR_104897 |
| *Leifsonia xyli* | 97.45 | 2.53E-124 | NR_074130 |
| *Lentibacillus salinarum* | 96.7 | 4.92E-127 | NR_044305 |
| *Lentibacillus salis* | 93.77 | 3.54E-116 | NR_043170 |
| *Loriellopsis cavernicola* | 87.18 | 2.10E-87 | NR_117881 |
| *Methylobacterium longum* | 98.54 | 1.51E-133 | NR_117045 |
| *Modestobacter marinus* | 98.9 | 3.54E-135 | NR_116228 |
| *Mycoplasma auris* | 78.16 | 2.23E-55 | NR_026035 |
| *Oceanobacillus pacificus* | 96.28 | 4.20E-95 | NR_126261 |
| *Opitutus terrae* | 92.67 | 2.24E-112 | NR_074978 |
| *Ornithinibacillus scapharcae* | 96.34 | 7.27E-125 | NR_117927 |
| *Paenibacillus cellulositrophicus* | 94.86 | 3.80E-89 | NR_116564 |
| *Paenibacillus taiwanensis* | 94.12 | 2.90E-117 | NR_044007 |
| *Paucisalibacillus globulus* | 97.8 | 2.23E-131 | NR_042445 |
| *Pedobacter saltans* | 90.11 | 1.32E-102 | NR_074586 |
| *Pseudomonas aeruginosa* | 99.63 | 6.83E-138 | NR_118644 |
| *Robinsoniella peoriensis* | 96.34 | 5.99E-126 | NR_041882 |
| *Romboutsia ilealis* | 95.35 | 1.98E-106 | NR_125597 |
| *Rubellimicrobium aerolatum* | 95.6 | 3.10E-123 | NR_044469 |
| *Shewanella baltica* | 99.27 | 1.01E-135 | NR_074843 |
| *Sporosarcina luteola* | 96.7 | 4.92E-127 | NR_114283 |
| *Sporosarcina pasteurii* | 98.53 | 9.50E-130 | NR_104923 |
| *Terrisporobacter mayombei* | 98.11 | 5.59E-120 | NR_104744 |
| *Thermoactinomyces intermedius* | 98.53 | 4.31E-134 | NR_041760 |
| *Tissierella creatinophila* | 95.38 | 5.00E-114 | NR_037028 |
| *Tissierella praeacuta* | 94.85 | 1.23E-115 | NR_044860 |
| *Turicibacter sanguinis* | 94.14 | 3.77E-122 | NR_028816 |
| *Virgibacillus campisalis* | 97.07 | 1.16E-128 | NR_108840 |
| *Virgibacillus dokdonensis* | 98.9 | 3.54E-135 | NR_043206 |
| *Virgibacillus halotolerans* | 98.9 | 3.54E-135 | NR_108860 |
| *Virgibacillus pantothenticus* | 96.73 | 8.89E-124 | NR_114091 |
