# Supplementary material for: Gut Microbiome of an 11th Century A.D. Pre-Columbian Andean Mummy
Source: PLoS One. 2015 Sep 30;10(9):e0138135. doi: 10.1371/journal.pone.0138135 (PMC4589460; doi:10.1371/journal.pone.0138135)
Supplement: S6 Table — (DOCX) [file pone.0138135.s022.docx]

**Supplemental Table 6.** BLASTn analyses of mummy’s microbiome (descending colon) against available *Trypanosoma cruzi* strains.

| ***Trypanosoma cruzi* strain** | **Best hit (Region)** | **Length (bp)** | **Identity (%)** | **e-value** |
| --- | --- | --- | --- | --- |
| **CL Brener** | gi\|70851345\|gb\|AAHK01016596.1\| | 111 | 89 | 1.0E-31 |
|  | gi\|70848846\|gb\|AAHK01019093.1\| | 124 | 86 | 2.0E-30 |
|  | gi\|70863139\|gb\|AAHK01005109.1\| | 299 | 83 | 1.0E-69 |
|  | gi\|70859655\|gb\|AAHK01008360.1\| | 451 | 97 | 0.0E+00 |
|  | gi\|70861786\|gb\|AAHK01006336.1\| | 213 | 89 | 2.0E-69 |
|  | gi\|70858470\|gb\|AAHK01009511.1\| | 109 | 81 | 4.0E-15 |
|  |  |  |  |  |
| **Dm28c** | gi\|557859122\|gb\|AYLP01000175.1\| | 111 | 89 | 4.0E-32 |
|  |  |  |  |  |
| **Esmeraldo** | gi\|431948721\|gb\|ANOX01005600.1\| | 376 | 84 | 1.0E-94 |
|  | gi\|431948498\|gb\|ANOX01005823.1 | 366 | 90 | 2.0E-135 |
|  | gi\|431951988\|gb\|ANOX01002333.1\| | 111 | 89 | 5.0E-32 |
|  | gi\|431947418\|gb\|ANOX01006903.1\| | 237 | 96 | 2.0E-107 |
|  | gi\|431951991\|gb\|ANOX01002330.1\| | 109 | 81 | 2.0E-15 |
|  |  |  |  |  |
| **JR cl4** | gi\|440824831\|gb\|AODP01001459.1 | 111 | 89 | 6.0E-32 |
|  | gi\|440824830\|gb\|AODP01001460.1\| | 109 | 81 | 2.0E-15 |
|  |  |  |  |  |
| **Marinkellei** | gi\|407393785\|gb\|AHKC01020053.1\| | 111 | 89 | 5.0E-32 |
|  | gi\|407393131\|gb\|AHKC01020501.1\| | 222 | 86 | 7.0E-59 |
|  | gi\|407391072\|gb\|AHKC01022161.1\| | 310 | 82 | 2.0E-70 |
|  | gi\|407403440\|gb\|AHKC01013149.1\| | 109 | 81 | 1.0E-15 |
|  |  |  |  |  |
| **Sylvio** | gi\|407831022\|gb\|ADWP02026601.1\| | 111 | 89 | 3.0E-15 |
|  | gi\|407840309\|gb\|ADWP02019721.1\| | 222 | 86 | 8.0E-59 |
|  | gi\|407855425\|gb\|ADWP02010818.1\| | 310 | 82 | 2.0E-70 |
|  | gi\|407830647\|gb\|ADWP02026919.1\| | 109 | 81 | 2.0E-15 |
|  |  |  |  |  |
| **Tula cl2** | gi\|478877473\|gb\|AQHO01016241.1\| | 131 | 90 | 3.0E-42 |
|  | gi\|478881375\|gb\|AQHO01012339.1\| | 738 | 80 | 2.0E-142 |
|  | gi\|478878127\|gb\|AQHO01015587.1\| | 132 | 82 | 4.0E-24 |
|  | gi\|478881319\|gb\|AQHO01012395.1\| | 726 | 90 | 0.0E+00 |
|  | gi\|478880269\|gb\|AQHO01013445.1\| | 496 | 84 | 2.0E-132 |
|  | gi\|478889933\|gb\|AQHO01003782.1\| | 111 | 89 | 1.0E-31 |
